# PBRM1 Deficiency Reshapes an Immune Suppressive Microenvironment Through Epigenetic Tuning of PBRM1‐KDM5C‐IL6 Axis in ccRCC

**DOI:** 10.1002/advs.202512627

**Published:** 2026-01-09

**Authors:** Wenjiao Xia, Hongru Wang, Yu Dong, Zitong Yang, Yiyang Zhou, Zhinan Xia, Qinchen Li, Liangliang Ren, Yichun Zheng, Junliang Yan, Dongmei Ma, Zhi Chen, Xingang Cui, Guixin Zhu, Cheng Zhang

**Affiliations:** ^1^ Department of Urology Center for Oncology Medicine, and International School of Medicine International Institutes of Medicine The Fourth Affiliated Hospital of School of Medicine Zhejiang University Yiwu China; ^2^ Department of Urology Xinhua Hospital Affiliated to Shanghai Jiaotong University School of Medicine Shanghai China; ^3^ Department of Urology The Fourth Affiliated Hospital of Harbin Medical University Harbin China; ^4^ Department of Urology, and International School of Medicine International Institutes of Medicine The Fourth Affiliated Hospital of School of Medicine Zhejiang University Yiwu China; ^5^ Department of Ultrasound and International School of Medicine International Institutes of Medicine The Fourth Affiliated Hospital of School of Medicine Zhejiang University Yiwu China; ^6^ Department of Anesthesiology, and International School of Medicine International Institutes of Medicine The Fourth Affiliated Hospital of School of Medicine Zhejiang University Yiwu China

**Keywords:** cancer‐associated fibroblasts, immunotherapy, PBRM1, tumor‐associated macrophages

## Abstract

*Polybromo 1 (PBRM1)* ranks as the second most commonly mutated gene in clear cell renal cell carcinoma (ccRCC), while its role in immune escape remains elusive. We developed a PBRM1‐knockout mice model to perform single‐cell RNA sequencing, which demonstrated a substantial population of immunosuppressive tumor‐associated macrophages (TAMs) in the spontaneous tumor, with consistent results from an orthotopic renal tumor mice model. Multiplex immunohistochemistry of clinical samples revealed that PBRM1‐deficient tumors exhibited increased M2 TAMs in both stroma and parenchyma, while CD8^+^ T cells were restricted to the stroma. M2 TAMs and cancer‐associated fibroblasts (CAFs) interacted to construct a tumor immune barrier, preventing CD8^+^ T cell infiltration. Mechanistically, PBRM1 modulated interleukin‐6 (IL‐6) expression by recruiting lysine demethylase 5C (KDM5C), thereby orchestrating M2 polarization of TAMs. Blocking IL‐6 synergistically augmented the antitumor efficacy of anti‐PD‐1 therapy. Our findings revealed a PBRM1‐KDM5C‐IL‐6 axis that influenced antitumor immunity, indicating a potential immunotherapeutic strategy in PBRM1‐deficient ccRCC.

## Graphical abstract

*PBRM1* ranks as the second most commonly mutated gene in ccRCC. This study reveals that PBRM1 loss promotes an immunosuppressive microenvironment by elevating M2 TAMs via the KDM5C–IL‐6 axis. These M2 TAMs, along with CAFs, form a barrier that excludes CD8^+^ T cells. Targeting IL‐6 synergizes with anti‐PD1 therapy, offering a promising strategy for PBRM1‐deficient tumors.

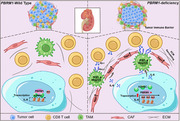

## 1 Introduction

Clear cell renal cell carcinoma (ccRCC) has long been acknowledged as an immunogenic tumor [1, 2, 3]. The treatment landscape for advanced ccRCC has evolved significantly with the approval of immune checkpoint inhibitors (ICIs) [4, 5, 6, 7]. However, the majority of patients continue to struggle with achieving a durable response to ICIs due to intrinsic or adaptive resistance [8, 9, 10, 11]. Emerging evidence has revealed that a non‐immunogenic tumor microenvironment (TME) poses a significant barrier to the efficacy of immunotherapy [12, 13]. Response to ICIs requires the presence of anti‐tumor T cells in the TME, while their activity is inhibited by checkpoint pathways [14, 15, 16]. In contrast to tumors with robust immunogenicity such as melanoma, ccRCC is characterized by a low to moderate tumor mutational burden (TMB) [17, 18]. Most ccRCC cases are associated with genetic deletions and mutations, or epigenetic silencing of the *von Hippel‐Lindau (VHL)* gene [19, 20]. Furthermore, ccRCC encompasses a spectrum of secondary mutations, including those in *Polybromo‐1 (PBRM1)*, *SET domain containing 2 (SETD2)*, and *BRCA1 associated protein 1 (BAP1)* [21]. Their contributions to immune modulation remain largely unresolved.

PBRM1, also known as BAF180, is part of the switch/sucrose non‐fermenting (SWI/SNF) chromatin remodeling complex [22, 23]. Existing findings regarding the impact of PBRM1 deficiency on immune responsiveness are still inconsistent. Recent studies established a connection between PBRM1 deficiency and improved clinical efficacy of anti‐PD‐1 therapy in ccRCC patients who previously underwent antiangiogenic therapy [4, 24]. Conversely, other contemporary research groups did not consider PBRM1 deficiency as a positive predictive biomarker for the response to ICIs in ccRCC [25, 26]. It has been observed that PBRM1‐deficient murine B16F10 melanomas were more immunogenic and exhibited increased responsiveness to immunotherapy [27]. Still, due to discrepancies in immunogenicity exhibited by various cancer types, the relevance of this conclusion to human ccRCC remains significantly constrained [28, 29, 30]. Therefore, the mechanisms by which PBRM1 deficiency modulates the TME and subsequently influences immune therapeutic response in ccRCC remain inadequately characterized, necessitating further investigation.

In our study, through comprehensive analysis of immune cell distribution and interplay within the TME of PBRM1‐deficient ccRCC, we identified that a significant proportion of PBRM1‐deficient ccRCC could be classified as an “immune excluded” phenotype. Within this context, M2 tumor‐associated macrophages (TAMs) exerted a critical influence in suppressing CD8^+^ T cells and facilitating the establishment of the tumor immune barrier (TIB). Mechanistically, we delineated the epigenetic framework through which PBRM1 regulates the expression of IL‐6, a pivotal cytokine that stimulates the M2 polarization of TAMs. Preclinical combination therapy with anti‐IL‐6 and anti‐PD‐1 demonstrated a remarkable antitumor efficacy. Taken together, our work clarified the essential role of PBRM1 in antitumor immunity, providing valuable insights for the immunotherapeutic strategies targeting PBRM1‐deficient ccRCC.

## 2 Results

### 2.1 *Vhl* ^f/f^ *Pbrm1* ^f/f^ *Ksp*‐*Cre* Mice Develop Multifocal ccRCC

**FIGURE 1:**
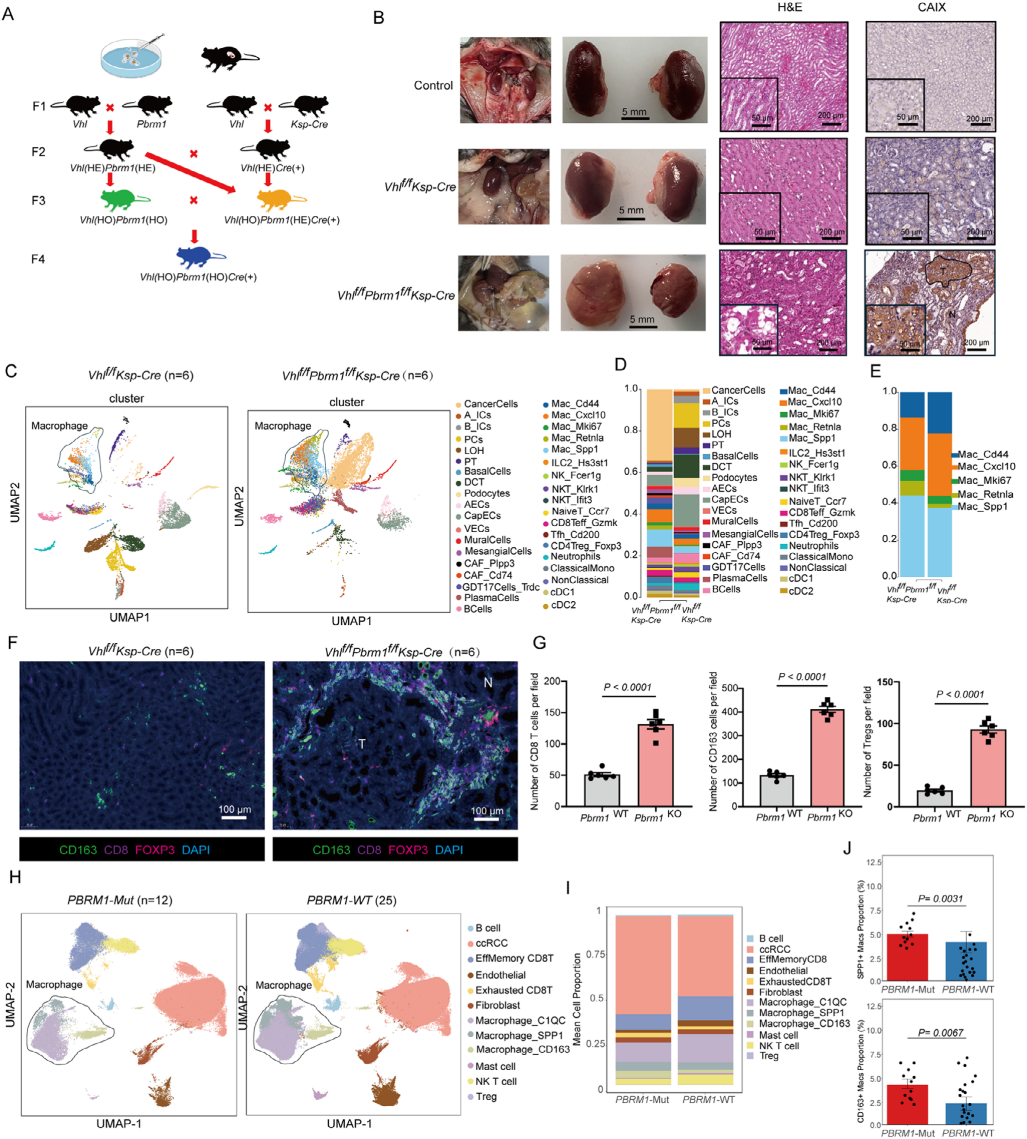
*Vhl* ^f/f^ *Pbrm1* ^f/f^ *Ksp*‐*Cre* mice develop multifocal ccRCC. (A) Schematic of crossings and resulting cohorts. HE, heterzygous; HO, homozygous. (B) Representative images from *Vhl^f/f^Pbrm1^f/f^Ksp‐Cre* mice showing enlarged nodular kidneys at 12 months of age compared to control mice and *Vhl^f/f^Ksp‐Cre* mice (left panel). Representative H&E (middle panel) and CAIX IHC (right panel) images of serial renal sections from control mice, *Vhl^f/f^Ksp‐Cre* mice and *Vhl^f/f^Pbrm1^f/f^Ksp‐Cre* mice of 12 months of age (*n* = 6 per group); scale bar, 5 mm. (C,D) UMAP representation and graph‐based clustering of merged scRNA‐seq data from all groups. (E) Macrophages cluster proportions and cell count. (F) Representative mIHC staining of kidneys from WT and *Vhl* ^f/f^ *Pbrm1* ^f/f^ *Ksp*‐*Cre* mice, green: CD163, red: Treg, purple: CD8, blue: DAPI (*n* = 6 per group); scale bar, 100 µm. (G) The column diagram showing the counts of spots with CD8^+^ T cells, CD163^+^ M2 TAMs and Tregs in WT and PBRM1 KO tumor slides. (H,I) UMAP representation and graph‐based clustering of merged scRNA‐seq data from all groups. (J) Proportions of SPP1^+^ TAMs and CD163^+^ TAMs among total TAMs in PBRM1 WT and PBRM1‐deficient ccRCC samples. Data presented as Mean ± SEM, unpaired two‐tailed Student's *t*‐test. (G,J). Data are representative of three independent experiments with similar results.

To explore the associations between *PBRM1* mutations and immune cell infiltration patterns within the TME, we first generated kidney‐specific *Pbrm1* and *Vhl* knockout mice and sought to establish a physiological mouse kidney cancer model that recapitulates human ccRCC (Figure 1A). As expected, the mRNA levels of VHL and PBRM1 were greatly reduced in the kidneys of *Vhl^f/f^Pbrm1^f/f^Ksp‐Cre* mice (Figure S1A). The PCR genotyping of DNA obtained from WT kidney or *Vhl^f/f^Pbrm1^f/f^Ksp‐Cre* tumor were correct (Figure S1B). Ultrasonic images of kidneys in *Vhl^f/f^Pbrm1^f/f^Ksp‐Cre* mice revealed a progression of imaging changes, starting from a normal appearance, advancing to cystic abnormalities, and eventually showing increased multifocal nodularity with a reduction in cystic features (Figure S1C). No renal tumors were observed in any of the aged control and *Vhl^f/f^Ksp‐Cre* animals. In contrast, *Vhl^f/f^Pbrm1^f/f^Ksp‐Cre* mice developed renal cancers by 12 months of age (Figure 1B). Double‐mutant mice displayed multifocal neoplastic tumors and he tumor cells exhibited key characteristics of human ccRCC, including clear cytoplasm and positive membranous staining of carbonic anhydrase IX (CA‐IX), a target of HIF‐1α (Figure 1B) [31, 32]. Deletion of *Vhl* and *Pbrm1* leads to HIF‐1α activation and increased expression of target genes, including *Glut1*, *Vegf*, *Pgk1*, *Hif1α* and *Pdk1* (Figure S1D). Finally, blood urea nitrogen (BUN) and creatinine levels were also significantly elevated in moribund *Vhl^f/f^Pbrm1^f/f^Ksp‐Cre* mice (Figure S1E,F), suggesting that these mice had impaired kidney function. Altogether, these data indicate that the combined loss of *Vhl* and *Pbrm1* within mouse tubular epithelia is sufficient for the development of renal neoplasias closely resembling the human disease, and PBRM1 functions as a tumor suppressor in ccRCC.

To provide detailed insights into the TME remodeling in *Pbrm1*‐deficient ccRCC, we conducted unbiased single‐cell RNA sequencing (scRNA‐seq) analysis on kidneys from 12‐month‐old *Pbrm1*‐proficient (WT) and *Pbrm1*‐deficient (KO) mice (Figure 1C). Expression profile analysis of 37 distinct cell clusters, with increased infiltration of GDT17s, Tregs and M2 TAMs (“Spp1^+^ Mac”, “Retnla^+^ Mac”) in tumors with *Pbrm1* deletion (Figure 1D,E). The proportions of other cell clusters within the total immune cell population were comparable between the WT and KO groups. Furthermore, multiplex immunohistochemistry (mIHC) staining uncovered that CD8‐positive and CD163‐positive cells were clustered around the tumors, with a significantly higher number of double‐positive cell pairs observed in the KO group (Figure 1F,G). To investigate whether altered PBRM1 levels are associated with a shift in TME immune landscapes in ccRCC. To further investigate the differences in the TME of PBRM1‐deficient human ccRCC, we collected both PBRM1 WT and PBRM1‐deficient ccRCC samples and performed scRNA‐seq. The scRNA‐seq results revealed that, compared to PBRM1 WT ccRCC, PBRM1‐deficient ccRCC exhibited a significant increase in the proportions of both SPP1^+^ TAMs and CD163^+^ TAMs, along with a higher percentage of exhausted T cells (Figure 1G,H). This further indicates an upregulation of M2‐type TAMs in PBRM1‐deficient ccRCC. These results demonstrated that PBRM1 deficiency shifts the TME into a context with high infiltration of immunosuppressive cells.

### 2.2 Deletion of PBRM1 in ccRCC Promotes Tumor Growth in Immunocompetent but not Immunodeficient Mice

We examined the protein levels of PBRM1 in 7 RCC cell lines, together with HK‐2 immortalized renal epithelial cells (Figure S2A). Using CRISPR‐Cas9 technology, we genetically deleted the *PBRM1* gene in human and mouse RCC cell lines proficient in PBRM1 (786‐O, ACHN, and Renca), and we selected three PBRM1 KO clones that lacked PBRM1 protein expression (Figure S2B). PBRM1 knockout had no discernable effect on clonogenic potential of RCC cells in culture compared with their isogenic WT progenitors, except under low serum conditions, where PBRM1 KO cancer cells demonstrated enhanced clonogenic potential (Figure S2C). Cell proliferation rates between WT and PBRM1 KO groups were similar (Figure S2D).

**FIGURE 2:**
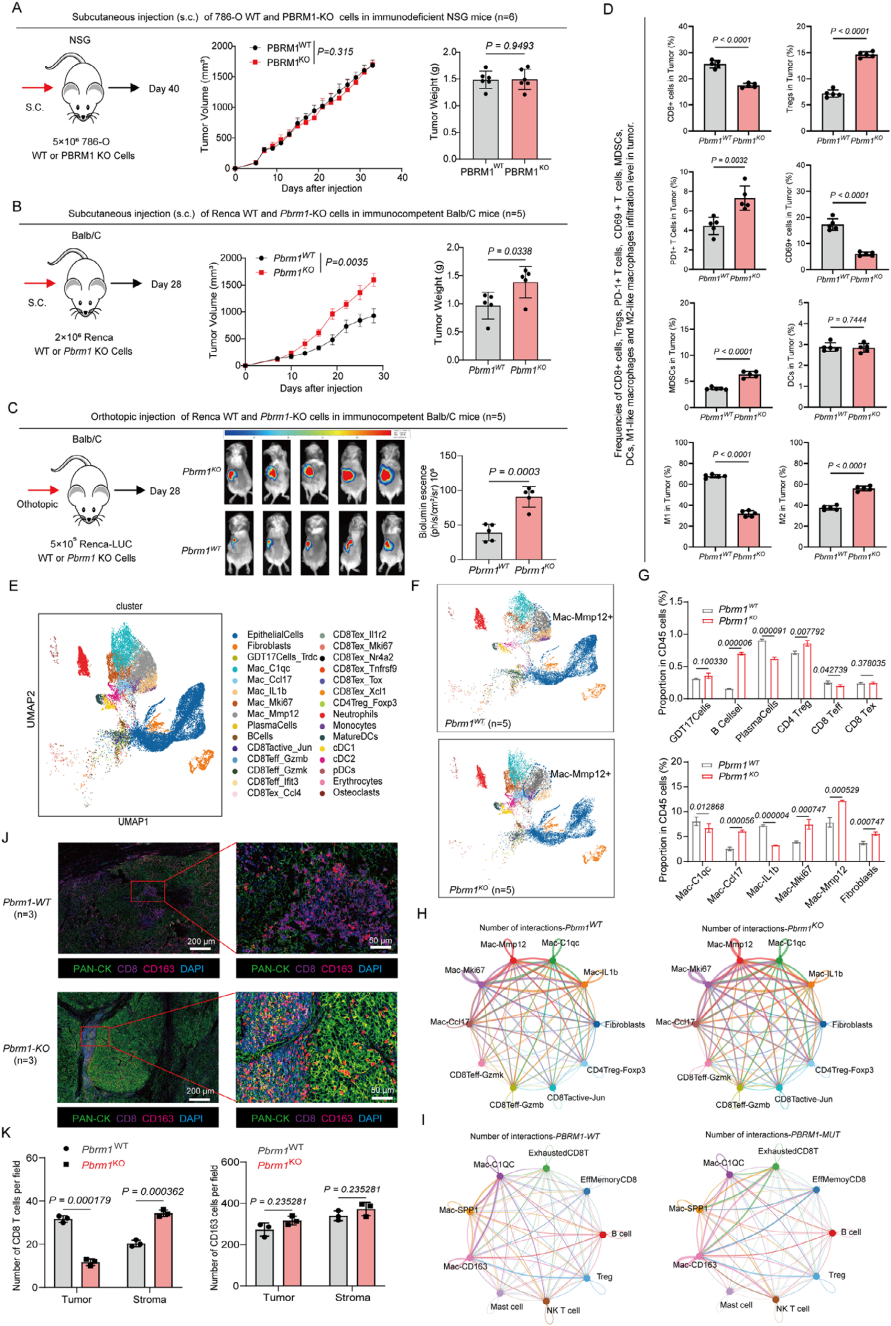
Deletion of PBRM1 in ccRCC promotes tumor growth in immunocompetent but not immunodeficient mice. (A) Schematic of the subcutaneous injection (s.c.) primary tumor growth assay after inoculation of 786‐O WT and PBRM1‐KO cells in immunodeficient NSG mice (*n* = 6 per group). On day 40, all mice were euthanized, and tumors were collected for further analysis. (B) Schematic of the subcutaneous injection (s.c.) primary tumor growth assay after inoculation of Renca WT and PBRM1‐KO cells in immunocompetent Balb/C mice (*n* = 5 per group). On day 28, all mice were euthanized, and tumors were collected for further analysis. (C) Schematic of the orthotopic inoculations primary tumor growth assay after inoculation of Renca WT and PBRM1‐KO cells in immunocompetent Balb/C mice (*n* = 5 per group). On day 28, all mice were euthanized, and tumors were collected for further analysis. (D) FACS analysis of harvested WT and PBRM1 KO tumors from subcutaneous tumors in the immunocompetent Balb/C mice showing the immune cells proportion (*n* = 5 per group). (E) UMAP representation and graph‐based clustering of merged scRNA‐seq data of all CD45^+^ cells from orthotopic tumor. (F) UMAP representation and graph‐based clustering of merged scRNA‐seq data of all CD45^+^ cells from orthotopic tumors in the WT (up panel) and PBRM1 KO (down panel) groups (*n* = 5 per group). (G) Proportion of each cell cluster among CD45^+^ cells in the 2 groups. (H) Cell‐cell interactions among immune cell types in mouse renal tumor tissues. (I) Cell‐cell interactions among immune cell types in human renal tumor tissues. (J)Representative mIHC staining of WT and PBRM1 KO tumors from subcutaneous tumors in the immunocompetent Balb/C mice (*n* = 3 per group), green: PAN‐CK, red: CD163, purple: CD8, blue: DAPI; scale bar, 200 µm. The image is representative from analysis of tumor specimens from three different mouse tumors. (K) The column diagram showing the counts of spots with CD8^+^ T cells and CD163^+^ M2 TAMs for infiltrated site in WT and PBRM1 KO tumor slides. Data presented as Mean ± SEM. Unpaired two‐sided Student's *t*‐test was used in (A–E) and one‐way ANOVA was used in (G,K). Data are representative of three independent experiments with similar results.

To probe the functional effects of PBRM1 KO on tumor phenotypes in vivo, WT and PBRM1 KO cell lines were inoculated in parallel into syngeneic and immunodeficient mice. Orthotopic and subcutaneous inoculations were used to assess tumor growth. Unexpectedly, distinct phenotypic differences emerged between tumors in immunodeficient and immunocompetent mice bearing these experimental primary tumors. In immunodeficient NSG mice, primary tumor growth was similar between WT and PBRM1 KO groups (Figure 2A and Figure S2E). However, in stark contrast, PBRM1 KO cancer cells exhibited significantly increased primary tumor growth (Figure 2B,C and Figure S2F,G) when inoculated into syngeneic, immunocompetent Balb/C mice. Collectively, these findings reveal that tumors derived from PBRM1 KO cancer cells grew more quickly in immunocompetent but not in immunodeficient mice, which suggested an involvement of the immune system. We further assessed the phenotype of immune cells by FACS from both WT and PBRM1 KO tumors (Figure S3). Their contrasting levels of expression suggested that KO tumors had less of CD69^+^/CD8^+^ T cells, CD86^+^ M1 TAMs and more abundant PD1^+^/CD8^+^ T cells, Tregs and CD206^+^ M2 TAMs (Figure 2D). To investigate whether altered PBRM1 levels are associated with a shift in TME immune landscapes in ccRCC, we carried out scRNA‐seq on CD45^+^ cells isolated from eight specimens, including both WT and PBRM1 KO orthotopic tumors. After quality control and doublet removal of scRNA‐seq data (see Method and Figure S4A), a total of 58037 cells were used for subsequent analysis (Figure 2E). Using published cell type‐specific gene signatures [33, 34], clusters with similar expression patterns were annotated, which revealed marked differences in the cellular constitution of WT vs. PBRM1 KO tumors (Figure 2F). We found that the proportions of mononuclear phagocytes (MPs) were significantly increased in PBRM1 KO tumors, to further explore the heterogeneity of MPs in PBRM1 KO tumors, we applied type specific markers that categorized 12031 cells into six subtypes, including Macrophages, Monocytes, Mature dendritic cells (Mature DCs), Conventional type 1 dendritic cells(cDC1), Conventional type 2 dendritic cells(cDC2) and Osteoclasts (Figure S4B). Next, we applied specific markers to categorize macrophages into 5 subtypes (Figure S4C), including (1) MMP12^+^ Mac with the function of promoting blood vessel and metabolism and extracellular matrix remodeling; (2) C1qc^+^ Mac with proinflammatory phenotype; (3) Mki67^+^ Mac with proliferative proinflammatory phenotype; (4) IL1b^+^ Mac and (5) immunomodulatory Ccl17^+^ Mac. We found the PBRM1 KO tumors were largely devoid of CD8^+^ T cells while M2 TAMs (MMP12^+^ Mac, Mki67^+^ Mac and Ccl17^+^ Mac) were largely increased (Figure 2G and Figure S4D). Notably, the macrophages infiltrating the PBRM1 KO tumors expressed markers indicative of their function of promoting blood vessel formation and metabolism and extracellular matrix (ECM) remodeling (Figure S4E–G). To further compare the differences in interaction patterns between macrophages and various cell types across the two groups, we analyzed the intercellular communication among several classes of immune cells. The results revealed that the most extensive interactions occurred between macrophages and CD8^+^ T cells in both mouse and human renal tumor tissues. In PBRM1 KO mouse tumors, communications between M2 TAMs (MMP12^+^ Mac, Mki67^+^ Mac and Ccl17^+^ Mac) and CD8^+^ T cells were significantly increased (Figure 2H). Similarly, in human PBRM1‐deficienct ccRCC, interaction strength between M2 TAMs (CD163^+^ Mac, SPP1^+^ Mac) and CD8^+^ T cells also showed an increasing trend (Figure 2I). These findings suggest that in PBRM1‐deficient ccRCC, M2‐like macrophages engage in immunosuppressive interactions with T cells, contributing to an immunosuppressive TME. In addition, mIHC staining demonstrated that CD8‐positive and CD163‐positive cells were closely situated around the tumors, and the number of double‐positive cell pairs was significantly higher in PBRM1 KO than in WT tumors (Figure 2J,K), suggesting that potential crosstalk between these two cell types contributed to the formation of the TIB structure associated with immunotherapy efficacy.

### 2.3 PBRM1 Deficiency Promotes Contacts Between CD8^+^ T Cells and M2 TAMs in Tumor Stroma and Reduces the Infiltration of CD8^+^ T Cells in the TME

**FIGURE 3:**
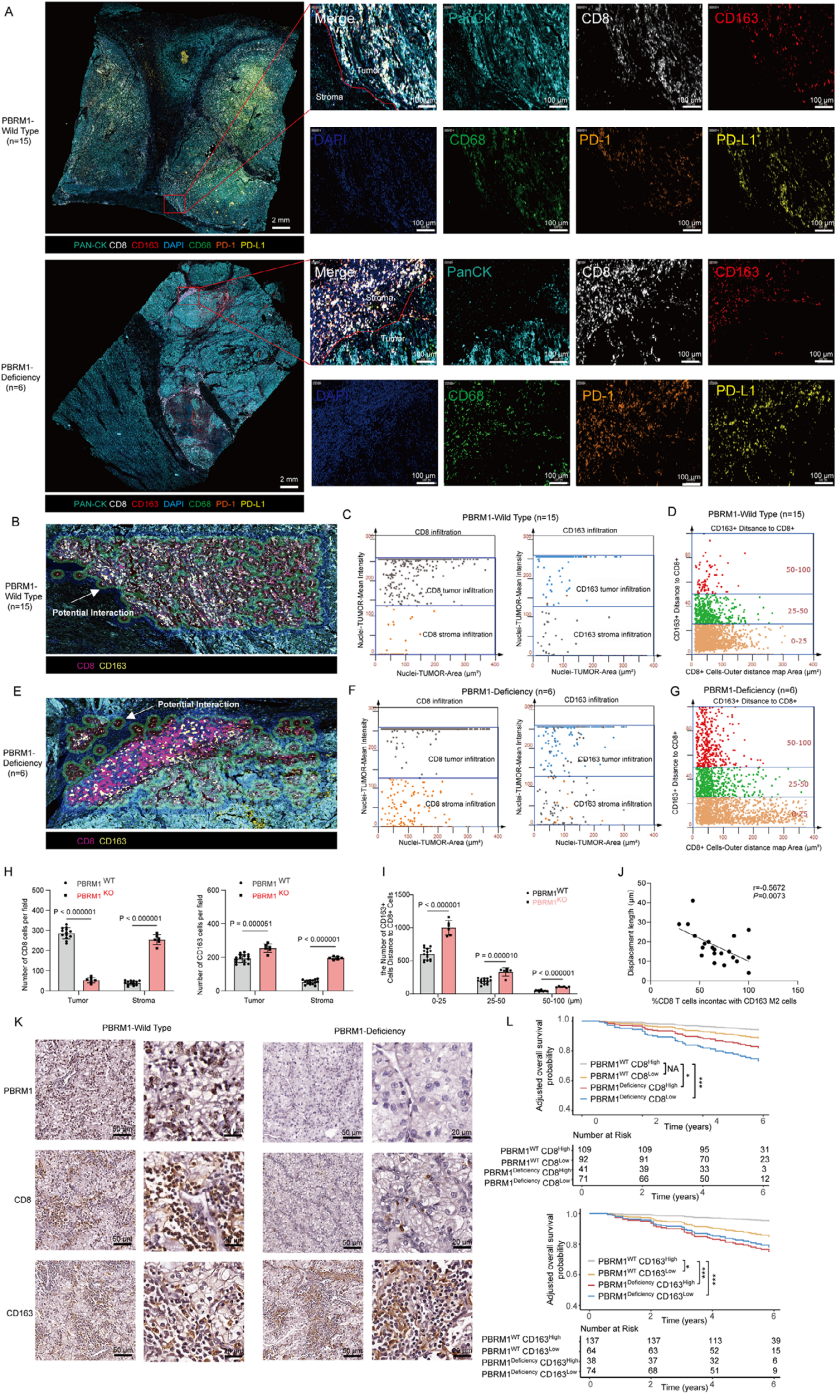
PBRM1 deficiency promotes CD8^+^ T Cells contacts with M2 TAMs in tumor stromal and reduce the infiltration of CD8^+^ T Cells in the TME. (A) Representative mIHC staining of PBRM1‐WT (up panel) (*n* = 15 per group) and PBRM1‐deficient ccRCC tissues (down panel) (*n* = 6 per group), blue‐green: PAN‐CK, bright: CD8, red: CD163, green: CD68, orange: PD‐1, yellow: PD‐L1, blue: DAPI; scale bar, 2 mm and 100 µm. The image is representative from analysis of tumor specimens from six different patient tumors. (B) Representative multi‐color staining of PBRM1‐WT ccRCC tissues from A (up panel) (*n* = 15 per group), purple: CD8, yellow: CD163. The circularly marked regions in the figures indicate areas where CD163 cells and CD8 cells are spatially close to one another suggesting potential interactions between them. (C) The flow‐like cytometer plots showing the mean identify of tumor‐infiltrating CD8^+^ T/CD163^+^ TAMs and stroma‐infiltrating CD8^+^ T/CD163^+^ TAMs in PBRM1‐WT ccRCC tissues. (D) The spatial distribution of CD163^+^ TAMs within the distance gradients of gradients of CD8^+^ T cells (0–25 µm, 25–50 µm, 50–100 µm, and 100–150 µm) in PBRM1‐WT ccRCC tissues. (E) Representative multi‐color staining of PBRM1‐deficient ccRCC tissues from A (down panel) (*n* = 6 per group), purple: CD8, yellow: CD163. The circularly marked regions in the figures indicate areas where CD163 cells and CD8 cells are spatially close to one another suggesting potential interactions between them. (F) The flow‐like cytometer plots show the mean identify of tumor‐infiltrating CD8^+^ T/CD163^+^ TAMs and stroma‐infiltrating CD8^+^ T/CD163^+^ TAMs in PBRM1‐deficient ccRCC tissues. (G) The spatial distribution of CD163^+^ TAMs within the distance gradients of gradients of CD8^+^ T cells (0–25 µm, 25–50 µm, 50–100 µm, and 100–150 µm) in PBRM1‐deficient ccRCC tissues. (H) Summary data for the expression of CD8^+^ cells and CD163^+^ cells in tumor and stroma. (I) Summary data for the spatial distribution of CD163^+^ TAMs within the distance gradients of gradients of CD8^+^ T cells. (J) Displacement length of endogenous CD8^+^ T cells in the stroma as a function of the percentage of CD8^+^ T cells in contact with TAMs in the same region for each microscopic field. (K) Representative IHC staining images of PBRM1, CD8 and CD163 in PBRM1‐WT and PBRM1‐deficient ccRCC tissues. Scale bar: 50 and 20 µm. (L) Adjusted survival curves comparing the prognostic differences between various groups with co‐expression of PBRM1/CD8 and PBRM1/CD163. Univariate Cox regression analysis was performed using the coxph() function from the R package Survival to evaluate the prognostic impact of age, gender, clinical stage, and pathological grade. A model was constructed and corrected survival curves were plotted using the Survival, survminer, and adjusted Curves R packages. Data are represented as Mean ± SEM. A one‐way ANOVA test in (H,I). Pearson correlation test (J). Data are representative of three independent experiments with similar results.

In both PBRM1‐WT and PBRM1‐deficient ccRCC patients, we analyzed paraffin‐embedded tissue sections to obtain insights into the localization of CD8^+^ T cells relative to tumor islets (stained by cytokeratin) and the surrounding stroma. Using mIHC, we explored the distribution of the main immune cell populations in both PBRM1‐WT and PBRM1‐deficient ccRCC specimens, and noted that CD8^+^ T cells were predominantly enriched in the stroma compared to tumor nests in PBRM1‐deficient ccRCC (Figure 3A). The cytometry panoramic tissue quantification assay developed by Li et al. [35], known as TissueFAXS, was utilized to elucidate the distinct spatial roles of CD163^+^ TAMs and CD8^+^ T cells in PBRM1‐WT (Figure 3B–D) and PBRM1‐deficient ccRCC tissues (Figure 3E–G). Figure 3B,E are selected from random fields in Figure 3A, with the selection criteria being the inclusion of both tumor and stromal areas. For each mIHC sample, three random fields were chosen, and the analysis was performed using TISSUEGENOSTICS software. Our primary focus was on CD163^+^ cells and CD8^+^ cells. According to the software analysis, cells located within 25 µm of each other were considered to potentially interact (as indicated by arrows in Figure 3B,E). Tumor and stromal areas were distinguished based on Pan‐CK staining, and CD163^+^ and CD8^+^ cells were localized and analyzed. The results showed that in PBRM1 WT ccRCC, the number of tumor‐infiltrating CD8^+^ cells was higher in the tumor compared to the stroma, whereas in PBRM1‐deficient ccRCC, the majority of CD8^+^ cells were localized in the stroma (Figure 3C,F,H). In PBRM1‐deficient ccRCC, both tumor‐infiltrating CD163^+^ cells and stromal CD163^+^ cells were more numerous than in PBRM1 WT ccRCC (Figure 3C,F,H). Based on the distance from the Pan‐CK positive area (0–25 µm, 25–50 µm, 50–100 µm), the number of CD163^+^ cells was counted, and a scatter plot was created based on their proximity to CD8^+^ cells. The results demonstrated that in PBRM1‐deficient ccRCC, the number of CD163^+^ cells was significantly higher than in PBRM1 WT ccRCC, and the interaction with CD8^+^ cells increased significantly at different distances (Figure 3D,G,I). This is further confirmed by the inverse relation that can be observed in these samples between the percentage of stromal CD8 T cells found in contact with a macrophage in the stroma (Figure 3J). We hypothesized that, together with other stromal components such as the ECM fibers and fibroblasts, M2 TAMs can reduce the motility of CD8^+^ T cells in the stroma, contributing to their confinement in this compartment and limiting their infiltration into tumor nests. We next examined whether the effect of PBRM1 deficiency on M2 TAMs has a profound impact on the clinical outcomes of patients with ccRCC. We analyzed data from the TCGA_KIRC cohort, grouping patients based on the presence or absence of PBRM1 mutations, and performed mutation frequency analysis (Figure S5A–L). We have established a clinical cohort comprising 313 ccRCC patients, the clinical and pathological characteristics of this cohort are presented in Table S1. As indicated by IHC staining, the expression of PBRM1 is positively correlated with the expression of CD8 in ccRCC tissues (Figure 3K and Figure S6A,B). We then conducted univariate Cox regression analysis on clinical pathological factors such as age, gender, clinical stage, and pathological grade for our cohort, and the results showed that, aside from gender, other clinical pathological factors all exhibited significantly higher hazard ratios (HR) (Tables S1 and S2). Consequently, in addition to the indicators of PBRM1, CD8, and CD163, we have further considered the impact of prognostic confounders such as age, stage, and grade. We constructed a model based on the Cox analysis and, after adjusting for the influence of clinical pathological factors, plotted the corrected survival curves. Survival analysis indicated that low PBRM1 expression is associated with low Overall Survival (OS). Notably, PBRM1^WT^/CD8^High^ patients experienced better OS than PBRM1^deficiency^/CD8^Low^ patients, and PBRM1^WT^/CD163 ^Low^ patients experienced better OS than PBRM1^deficiency^/CD163^High^ patients (Figure 3L and Figure S6C–F). Thus, high tumor infiltration and functional reprogramming of M2 TAMs induced by PBRM1 deficiency exert a negative impact on the prognosis of ccRCC patients.

### 2.4 PBRM1 Deficiency Promotes the Secretion of Tumor‐Derived IL‐6 and Enhances M2‐Like Polarization of Macrophages

**FIGURE 4:**
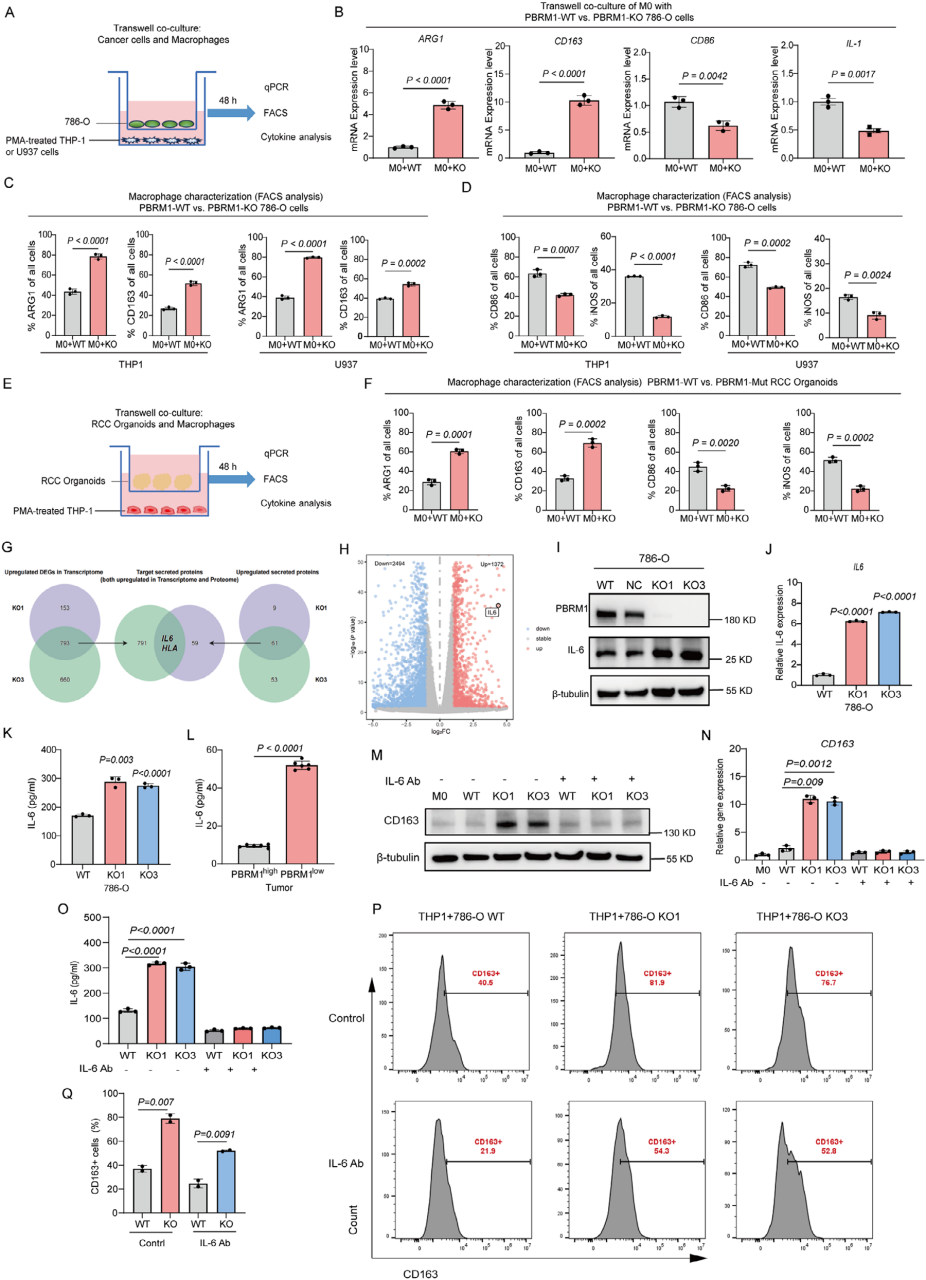
PBRM1 deficiency promotes the secrete of tumor‐derived IL‐6 and enhances M2‐like polarization of macrophages. (A) Schematic representation of a trans‐well coculture assay involving ex vivo programmed M0 macrophages combined with WT and PBRM1 KO cancer cells. (B) qRT‐PCR of mRNA expression *ARG1*, *CD163*, *CD86*, *IL‐1* in M0 macrophages cocultured with WT and PBRM1 KO cancer cells. (C) FACS analysis of ARG1 and CD163 (M2‐like marker) expression in M0 macrophages cocultured with WT and PBRM1 KO cancer cells. (D) FACS analysis of CD86 and iNOS expression (M1‐like marker) in M0 macrophages cocultured with WT and PBRM1 KO cancer cells. (E) Schematic representation of a trans‐well coculture assay involving ex vivo programmed M0 macrophages combined with WT and PBRM1 KO PDOs. (F) FACS analysis of M1‐like markers and M2‐like markers expressions in M0 macrophages cocultured with WT and PBRM1 KO PDOs. (G) Intersection of Venn diagram showed the upregulated genes and proteins in PBRM1 KO cancer cells. (H) RNA‐seq analysis of differential expressed genes in WT and PBRM1 KO cancer cells. (I) Western blot analysis of IL‐6 expression in WT and PBRM1 KO cancer cells. (J) qRT‐PCR analysis of IL‐6 expression in WT and PBRM1 KO cancer cells. (K) ELISA analysis of IL‐6 expression in WT and PBRM1 KO cancer cells. (L) ELISA analysis of IL‐6 expression in PRBM1‐WT and PBRM1‐deficient tumor tissues. (M) Western blot analysis of IL‐6 expression in WT and PBRM1 KO cancer cells with and without IL‐6 Ab treatment. (N) qRT‐PCR analysis of IL‐6 expression in WT and PBRM1 KO cancer cells with and without IL‐6 Ab treatment. (O) ELISA analysis of IL‐6 expression in WT and PBRM1 KO cancer cells with and without IL‐6 Ab treatment. (P) FACS analysis of CD163 protein expression in M0 macrophages cocultured with WT and PBRM1 KO cancer cells with and without IL‐6 Ab treatment. (Q) The column diagram showed the percent of CD163 protein expression in (P). N = 3 samples for each group. Data presented as Mean ± SEM, unpaired two‐tailed Student's t test (B, C, D, F, J, L) or one‐way ANOVA with Tukey's multiple comparisons tests (J, K, N, O, Q). Data are representative of three independent experiments with similar results.

To better understand the effect of PBRM1 on macrophages, we used a co‐culture system to link trafficking between ccRCC cells and macrophages (Figure 4A and Figure S7A). We found that THP‐1(or U937)‐differentiated macrophages co‐cultured with PBRM1 KO cancer cells increased the expression of M2‐like markers (such as CD163 and ARG1), while the transcription of M1‐like markers (such as CD86 and IL‐1), was decreased (Figure 4B). FACS analysis showed that the M0 macrophages co‐cultured with PBRM1 KO cancer cells from the trans‐well assay exhibited a CD86^low^ iNOS^low^/ARG1^high^ CD163^high^ phenotype, compared with those co‐cultured with WT cancer cells (Figure 4C,D). Another trans‐well coculture assay was established (Figure S7B) to assess the effects of WT vs PBRM1 KO cancer cells on bone marrow‐derived macrophages (BMDMs). PBRM1 KO cancer cells induced the expression of ARG1 and CD163, which were prototypically expressed in M2‐like macrophages (Figure S7D,E), whereas WT cancer cells induced the expression of CD86 and iNOS in M1‐like macrophages (Figure S7F,G). Furthermore, we isolated primary cells from PBRM1‐WT and PBRM1‐deficient patient samples and established patient‐derived organoid (PDO) models. The results confirmed the successful generation of organoids and no significant morphological differences observed in two groups (Figure S7C). Subsequently, we used a co‐culture system in which the organoids were co‐cultured with M0 macrophages derived from THP‐1 monocytes. Cells from the lower chamber were collected and analyzed by flow cytometry to determine macrophage M1/M2 polarization. The results demonstrated that, compared to co‐culture with PBRM1‐WT PDOs, co‐culture with PBRM1‐deficient PDOs led to a significant upregulation of M2 phenotypic markers (CD163, ARG1) and a downregulation of M1 phenotypic markers (CD86, iNOS) in macrophages (Figure 4E,F). Collectively, these bioassays revealed that PBRM1 KO cancer cells were differentially active in their capability to reprogram macrophages into an M2‐like immunosuppressive phenotype, consistent with the distinctive immune deserts that characterized PBRM1 KO tumors. These findings suggested that PBRM1‐deficiency promotes the migration and M2‐like polarization of macrophages in ccRCC.

To dissect the mechanism underlying the effect of PBRM1 on macrophages, we collected the supernatants from WT and PBRM1 KO cancer cells and detected the difference in protein content using Mass Spectrum (MS) (Table S3). Then, combining these results with differential expression of RNA‐seq genes in WT and PBRM1 KO cancer cells (Table S4), we identified IL‐6 as a significantly upregulated gene in PBRM1 KO cancer cells by intersection of Venn diagram (Figure 4G,H and Table S5). Western Blot, quantitative real‐time PCR (qRT‐PCR) and enzyme‐linked immunosorbent assay (ELISA) confirmed the increased IL‐6 expression and secretion in PBRM1 KO cancer cells compared with their counterparts (Figure 4I–K). Additionally, we observed increased levels of IL‐6 in the PBRM1‐deficient ccRCC tissues (Figure 4L). After adjusting for prognostic confounders such as age, stage, and grade, we plotted the survival curves for patients with high and low IL6 expression. The results showed that higher IL‐6 expression levels were associated with poorer OS and Disease‐Free Survival (DFS) in ccRCC patients (Figure S8A–D and Table S5). We categorized patient tumors into PBRM1 truncating mutations and PBRM1 missense mutations. In survival analysis, patients with truncating mutations showed a trend toward shorter progression‐free survival compared to the wild‐type group (*p* = 0.08), whereas the missense subgroup did not demonstrate a significant survival difference (Figure S8E). Correlation with tumor IL‑6 levels shows that samples with truncating mutations exhibit significantly higher IL‑6 expression compared to missense‐mutant tumors (*p* < 0.05) (Figure S8F). Nonetheless, the data suggest that loss‐of‐function (truncating) mutations, rather than missense variants, are more strongly associated with elevated IL‑6 and may correlate with more aggressive disease behavior. In addition, the IL6R expression in tumors was positively associated with CD163 expression (Figure S8G). We next investigated whether IL‐6 signaling mediates the effect of PBRM1 deficiency on macrophages. Blocking tumor‐derived IL‐6 signaling using an IL‐6 neutralizing antibody significantly reduced the M2‐like polarization of macrophages induced by PBRM1 depletion (Figure 4N–Q). Hence, we propose that tumor‐derived IL‐6 signaling is the key mediator of the PBRM1 deficiency induced immunosuppressive phenotype of macrophages.

### 2.5 PBRM1 Deficiency Results in Gained Histone H3 Lysine 4 Trimethylation (H3K4me3) Peaks and Inducing IL‐6 Transcription and Secretion in ccRCC

**FIGURE 5:**
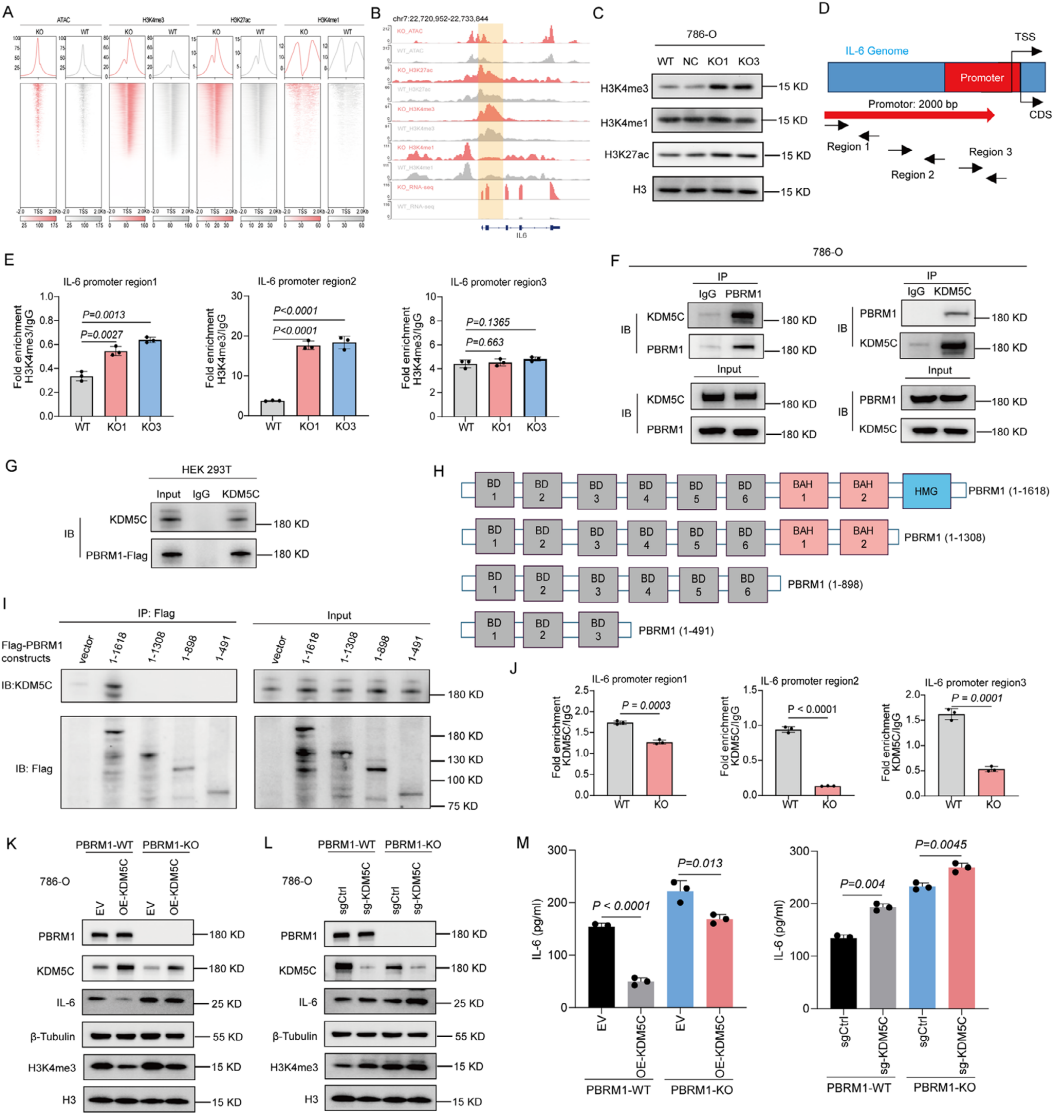
PBRM1 deficiency results in gained H3K4me3 peaks independently of open chromatin and induces IL‐6 transcription and secretion in ccRCC. (A) Heatmap of H3K4me3, H3K27ac and H3K4me1 peaks in 786‐O PBRM1 KO cells. (B) H3K4me3 ChIP‐seq track showing an example of a gained H3K4me3 peak in both PBRM1 KO lines (red) compared to the control line (gray). Heatmap illustrating mRNA levels and fold change of IL‐6 depositions in promoter. (C) Western blot analysis of H3K4me3 expression in WT and PBRM1 KO cancer cells. (D,E) ChIP assays to assess the degree of methylation of H3K4me3 within the regions 1–3 of the IL‐6 promoter in WT and PBRM1 KO cancer cells. (F) 786‐O cells harvested for immunoprecipitation. Immunoprecipitants and inputs were analyzed by immunoblots. (G) HEK293T cells were transfected with vector or Flag‐PBRM1 and harvested for immunoprecipitation with Flag beads and elution with 3× Flag peptide. Immunoprecipitants and inputs were analyzed by immunoblots. (H) Schematic depiction of the functional domains of PBRM1 and truncated constructs. WT: wild‐type, BAH: bromo‐adjacent homology domain, HMG: high‐mobility group domain. (I) HEK293T cells were transfected with vectors, Flag‐WT PBRM1 or Flag‐PBRM1 truncated constructs for immunoprecipitation with anti‐Flag beads, immunoprecipitants and inputs were analyzed by immunoblots. (J) ChIP assays to assess the degree of KDM5C within the regions 1–3 of the IL‐6 promoter in WT and PBRM1 KO cancer cells. (K) (L)Western blot analysis of IL‐6 and H3K4me3 expressions in different cells. (M) ELISA analysis of IL‐6 expression in different cells. N = 6 samples for each group. Data presented as Mean ± SEM, two‐way ANOVA (E) (M), and unpaired two‐tailed Student's *t*‐test (J). Data are representative of three independent experiments with similar results.

Given that mutations in SWI/SNF subunits have previously been associated with alterations in the histone modification landscape [36, 37, 38], we then analyzed publicly available transposase‐accessible chromatin (ATAC‐seq) data and chromatin immunoprecipitation followed by sequencing (ChIP‐seq) data for H3K4me3, Histone H3 Lysine 4 Mono‐methylation (H3K4me1), and Histone H3 Lysine 27 Acetylation (H3K27ac) in both WT and PBRM1 KO cancer cells. The multi‐omics data show that PBRM1 loss‐of‐function induced subtle changes in chromatin accessibility landscape and a significant increase in H3K4me3 peaks, a marker of active and poised promoters (Figure 5A and Table S8). We identified 3540 H3K4me3 peaks gained upon PBRM1 KO, while 1,029 peaks were lost (Figure S8F). No other significant changes were found in other histone modification markers (H3K27ac, H3K4me1) (Figure 5B and Table S9). Moreover, we found that ATAC‐seq peak signals remained consistent at both gained and lost H3K4me3 peak regions following PBRM1 KO (Figure S8H). These findings indicated that the changes in H3K4me3 levels likely occur independently of chromatin accessibility, a phenomenon that has also been observed with other histone modifications.

Next, we examined H3K4me3 levels and IL‐6 expression changes in our multi‐omics data and observed a robust increase in H3K4me3 peaks at the IL‐6 promoter, along with a significant rise in RNA‐seq signals following PBRM1 KO in 786‐O cells (Figure 5B). The upregulation of H3K4me3 in PBRM1 KO cancer cells was confirmed by Western blot (Figure 5C). We then investigated whether PBRM1 influenced the methylation pattern of H3K4me3 within the IL‐6 promoter region. ChIP assays were performed to assess the level of H3K4me3 at three selected regions within the IL‐6 promoter (IL‐6 1, 2, and 3) in both WT and PBRM1 KO cancer cells (Figure 5D). Additional negative control experiments were conducted using non‐immune IgG. As shown in Figure 5E, no big differences in the patterns of immunoprecipitated methylated proteins were observed using primers specific for regions 3. In contrast, an increase in H3K4me3 events were detected within the proximal IL‐6 promoter region 1 and 2 in PBRM1 KO cancer cells (Figure 5E).

To explore the mechanism by which PBRM1 affects H3K4 methylation status, we used the Biological General Repository for Interaction Datasets (BioGRID) to detect proteins that might interact with PBRM1. Among the many molecules, those having the potential to bind with PBRM1 and to affect H3K4me3 levels were highlighted. Lysine Demethylase 5C (KDM5C), a histone demethylase that removes methyl groups from tri‐methylated lysine four on histone H3, were selected [39, 40]. To test whether there is an interaction between PBRM1 and KDM5C, co‐IP analyses showed that endogenous PBRM1 pulled down the endogenous KDM5C, and vice versa, in 786‐O cells (Figure 5F). Besides, overexpressed PBRM1 was also capable of interacting with KDM5C in HEK‐293T cells (Figure 5G). PBRM1 contains a high‐mobility group (HMG) DNA‐binding domain, two uncharacterized BAH domains, and six bromodomains (BRDs) [41, 42, 43]. To reveal the key domains for the PBRM1‐KDM5C interaction, we used a series of plasmids encoding the full length and three truncated mutants of PBRM1 to carry out co‐IP analysis (Figure 5H). As shown in Figure 5I, PBRM1 without HMG domain failed to interact with KDM5C, implying that the HMG domain was indispensable for KDM5C association. Hence, KDM5C bound with the HMG domain of PBRM1 to maintain H3K4 trimethylation levels. We next investigated whether PBRM1 affected the binding of KDM5C to the IL‐6 promoter region. As expected, PBRM1 KO weakened the enrichment of KDM5C on the IL‐6 promoter region (Figure 5J). To further investigate the mechanism, we generated KDM5C‐overexpressing and knockdown cells in both PBRM1‐WT and PBRM1‐KO cells. Western blot analysis revealed that in PBRM1‐WT cells, KDM5C overexpression downregulated H3K4me3 levels and suppressed IL6 expression. In contrast, in PBRM1‐KO cells, KDM5C overexpression reversed the downregulation of H3K4me3 and restored IL6 expression (Figure 5K). In PBRM1 WT cells, knockdown of KDM5C upregulated H3K4me3 and IL6, whereas in PBRM1 KO cells, KDM5C knockdown led to a further upregulation of H3K4me3 and IL6 (Figure 5L). ELISA analysis further confirmed these results (Figure 5M). Taken together, these systematic genetic manipulations demonstrate that alterations in KDM5C‐mediated H3K4me3 levels are a key upstream event directly regulating IL‐6 expression. PBRM1 loss appears to hijack this epigenetic regulatory axis by affecting KDM5C recruitment to drive sustained IL‐6 expression.

### 2.6 The Interaction Between Cancer‐Associated Fibroblasts (CAFs) and M2‐Like Macrophages is Associated with the Formation of the TIB Structure in PBRM1‐Deficient ccRCC

**FIGURE 6:**
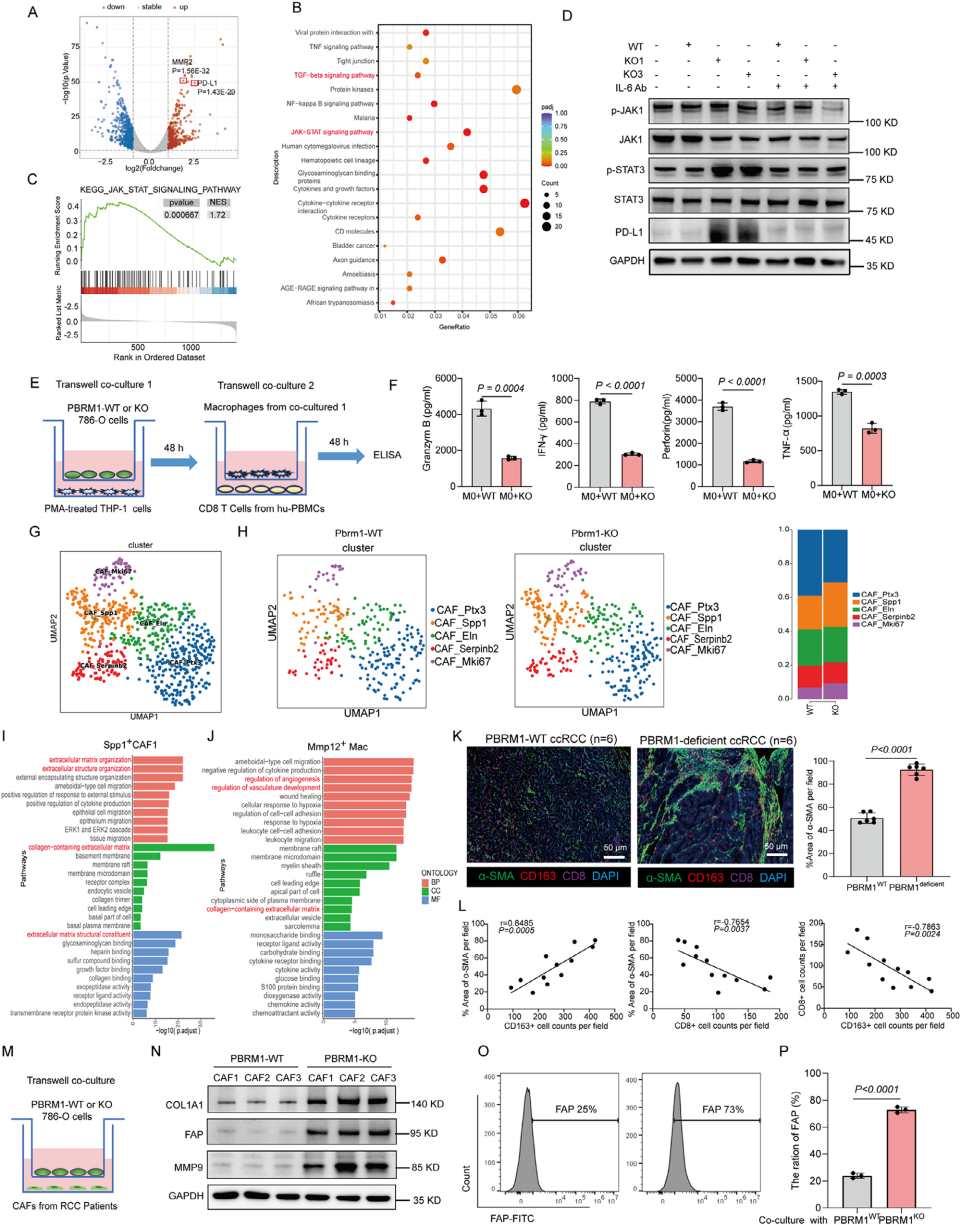
The interaction between CAFs and M2‐like macrophages is associated with the formation of the TIB structure in PBRM1‐deficient ccRCC. (A) Volcano plot of differentially expressed genes between M0 macrophages cocultured with WT and PBRM1‐KO cancer cells. (B) Gene ontology (GO) analysis of differentially expressed genes shown in panel A. (C) Pathway enrichment analysis of the upregulated genes in M0 macrophages cocultured with PBRM1 KO cancer cells. (D) Western blot analysis of the protein levels in M0 macrophages cocultured with WT and PBRM1 KO cancer cells. (E) Schematic representation of a trans‐well coculture assay involving ex vivo programmed M0 macrophages combined with WT and PBRM1 KO cancer cells and another trans‐well coculture assay with CD8^+^ T cells. (F) ELISA analysis of Granzym B, IFN‐γ, Perforin and TNF‐α expression in CD8^+^ T cells. (G) Re‐clustering of CAFs displayed in a UMAP plot, with circled five subclusters. (H) Relative cell proportion of CAFs subclusters in WT and Pbrm1‐deficient ccRCC. (I) GO enrichment of differentially expressed genes in spatial Spp1^+^ CAFs clusters in PBRM1 KO group. (J) GO enrichment of differentially expressed genes in spatial Mmp12^+^ macrophages clusters in PBRM1 KO group. (K) Representative mIHC staining and summary data of α‐SMA (green), CD163 (red) and CD8 (purple) from human PBRM1‐WT and PBRM1‐deficient ccRCC tissues; scale bar, 50 µm. N = 6 samples for each group. (L) Correlation between the expression levels of α‐SMA, CD8^+^ and CD163^+^ cells. (M) Schematic representation of a trans‐well coculture assay involving ex vivo CAFs combined with WT and PBRM1 KO cancer cells. (N) Western blot analysis of the Fibroblast‐associated markers levels in CAFs combined with WT and PBRM1 KO cancer cells. (O) FACS analysis of FAP protein expression in CAFs combined with WT and PBRM1 KO cancer cells. Data presented as Mean ± SEM, unpaired two‐tailed Student's *t*‐test (F, K, P), Pearson correlation test (L). Data are representative of three independent experiments with similar results.

To further identify the candidate biomarkers related to macrophages status, differential expression analysis was performed with macrophages cocultured with WT or PBRM1 KO cancer cells using bulk RNA‐seq. We identified 4400 differentially expressed genes (DEGs; FDR < 0.05, |log2FC| > 0.5) between macrophages cocultured with WT and PBRM1 KO cancer cells, among them, two up‐regulated genes, MMP‐2 and PD‐L1, gained our interest (Figure 6A). In addition, KEGG analysis demonstrated that co‐cultured with PBRM1 KO cancer cells could activate JAK‐STAT signaling pathway (Figure 6B,C). It has been reported that PD‐L1 expression in TAMs was associated with exhausted T cells [44, 45, 46]. To investigate whether PBRM1 regulated the expression of PD‐L1 in TAMs, we co‐cultured ccRCC cells with THP‐1 cells. Western blot showed that PD‐L1 was upregulated in THP‐1 cells, when co‐cultured with 786‐O PBRM1 KO cancer cells but not with WT cancer cells (Figure 6D). In addition, we found that PBRM1 deficiency induced M2 polarization and PD‐L1 expression of TAMs via activating JAK1/STAT3 pathway (Figure 6D).

Next, to explore whether PBRM1 deficiency affected the functions T cells, we co‐cultured the CD8^+^ T cells with WT or PBRM1‐KO induced macrophages (Figure 6E). It was worth noting that PBRM1‐KO induced macrophage not only inhibited the amount of IFN‐γ but also inhibited the expression of Granzym B, Perforin and TNF‐α in CD8^+^ T cells (Figure 6F). Since CD8^+^ T cells were enriched in the stroma relative to tumor nests in human PBRM1‐deficient tumors (Figure 3A), we hypothesized that this localization was in partially dictated by the density and organization of the ECM fibers, which can alter lymphocyte motility within the stroma. To prove this, we subclustered CAFs, a component of TIB, into five subtypes, each with unique signatures, based on the scRNA‐seq dataset of WT and PBRM1 KO groups (Figure 6G,H and Figure S9A). Then, we investigated the alteration of fibroblast cell subtypes between WT and PBRM1 KO groups and revealed that Spp1^+^ CAFs and Mki67^+^ CAFs were predominantly enriched in tumor tissues (Figure 6H). Furthermore, we identified several genes that highly expressed in spots enriched with Mmp12^+^ macrophages and Spp1^+^ CAFs, which contribute to TIB structure. Importantly, these genes were enriched in extracellular structure organization, ECM organization, and collagen‐containing ECM organization (Figure 6I,J). Consistently, functional enrichment analysis of genes specifically expressed in Mmp12^+^ macrophage and Spp1^+^ CAFs from the scRNA‐seq dataset revealed a shared KEGG pathway: ECM‐receptor interaction (Figure S9B), indicating that the colocalization of Mmp12^+^ macrophages and Spp1^+^CAFs might be related to the processes, such as cell migration, adhesion, and ECM organization. In addition, mIHC staining demonstrated that CD163^+^ M2‐like macrophages and αSMA^+^ CAFs were in close proximity around tumors, and the number of double‐positive cell pairs was significantly higher in PBRM1‐deficient tumors (Figure 6K,L and Figure S9C,D), suggesting that the potential crosstalk between these two cell types contributes to the formation of the TIB structure associated with immunotherapy efficacy. To validate this hypothesis, we isolated CAFs from ccRCC and co‐cultured them with PBRM1‐WT or PBRM1‐KO tumor cells in vitro (Figure 6M). As shown in Figure 6N, expressions of collagen type I alpha 1 chain (COL1A1, a major component of ECM), fibroblast activation protein (FAP), and matrix metalloproteinases 9 (MMP9) were higher in CAFs co‐cultured with PBRM1‐KO tumor cells. Consistent conclusions were further supported by flow cytometry and qPCR results (Figure 6O,P and Figure S9F). Given that TAMs and CAFs also contribute to IL‐6 production, we further investigated the primary cellular source of IL‐6 within the tumor microenvironment in our study. We isolated tumor cells, TAMs, and CAFs from patients with PBRM1‐WT and PBRM1‐deficient ccRCC. After counting, equal numbers of each cell type were plated, and the IL‐6 secretion level was measured using an ELISA kit. The results indicated that all three cell types were capable of secreting IL‐6, however, tumor cells secreted the highest amount. Furthermore, IL‐6 secretion was significantly upregulated in the PBRM1‐deficient group compared to the PBRM1‐WT group (Figure S9G). Together, these results indicated that MMP12^+^ macrophages and PD‐L1^+^ macrophages communicate with CAFs to facilitate T cells exclusion, and that the formation of the TIB structure might contribute to the immunosuppressive TME in PBRM1‐deficient ccRCC.

### 2.7 Targeting IL‐6 Destroys the TIB Structure and Sensitizes PBRM1‐Deficient ccRCC to Immunotherapy

**FIGURE 7:**
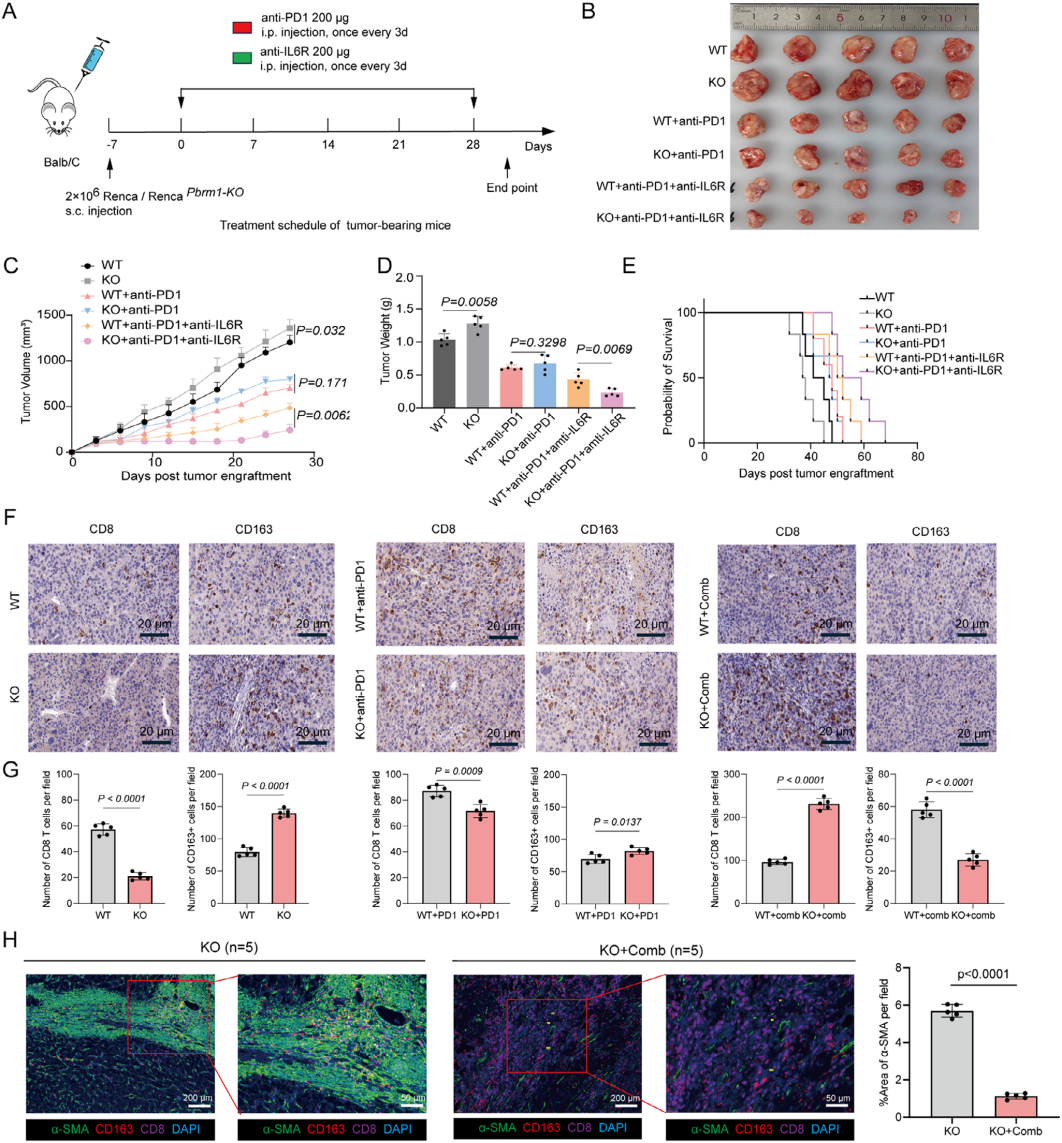
Targeting IL‐6 destroys the TIB structure and sensitizes PBRM1‐deficient ccRCC to immunotherapy. (A) Schematic showing the schedule of anti‐PD‐1 and anti‐IL‐6R treatment in mice bearing WT and Pbrm1 KO tumors. (B) Gross appearance of the tumors from the indicated treatment groups. (C) Tumor growth of the indicated treatment groups. (D) Tumor weight of the indicated treatment groups. (E) Kaplan–Meier survival analysis of the indicated treatment groups. (F) Representative IHC staining images of CD8 and CD163 in ccRCC tissues of each group. Scale bar: 20 µm. (G) Summary data for the expression of CD8^+^ cells and CD163^+^ cells in tumor. (H) Representative mIHC staining and summary data of α‐SMA (green), CD163 (red) and CD8 (purple) from subcutaneous tumors in Pbrm1 KO mice after anti‐IL‐6R and anti‐PD‐1combination therapy and control. Scale bar, 200 µm and 50 µm (*n* = 5). (H) Schematic diagram depicting the regulatory role of PBRM1 in the TME immune landscapes of ccRCC. N = 5 samples for each group. Data presented as Mean ± SEM, one‐way ANOVA (C,D), Log‐rank Mantel‐Cox test (E), unpaired two‐tailed Student's *t*‐test (G). Data are representative of at least two independent experiments.

Since the expression of IL‐6 was closely related to the immunosuppressive TME of PBRM1‐deficient ccRCC, blocking IL‐6/IL‐6R signaling may eliminate the inhibitory effect of M2 TAMs and CAFs on the immune microenvironment, thereby facilitating the infiltration of cytotoxic T cells into the tumor core. To test this possibility, we constructed subcutaneous ccRCC mice models with Pbrm1 KO cancer cells and administered either anti‐PD‐1 antibody or a combination of anti‐PD‐1 and anti‐IL‐6R to the mice on day 7 after tumor transplantation (Figure 7A). More significant tumor growth restriction was observed with the combination therapy compared to the control and monotherapies (Figure 7B–D). Notably, the combination of anti‐IL‐6R and anti‐PD‐1 dramatically increased the OS of Pbrm1‐deficient ccRCC bearing mice (Figure 7E).

IHC analysis showed that mice treated with combination therapy had more CD8^+^ T cells and less M2‐like macrophages infiltration into tumor tissues, suggesting that anti‐IL‐6R treatment shifts Pbrm1‐deficient ccRCC toward immunologically “hot” tumors, characterized by relatively high responses to ICIs (Figure 7F,G). The mIHC staining results revealed that the combination of anti‐IL‐6R and anti‐PD‐1 treatment markedly reduced the proportion of aSMA^+^ fibroblasts while increasing the proportion of CD8^+^ T cells in the tumor regions (Figure 7H).

**FIGURE 8:**
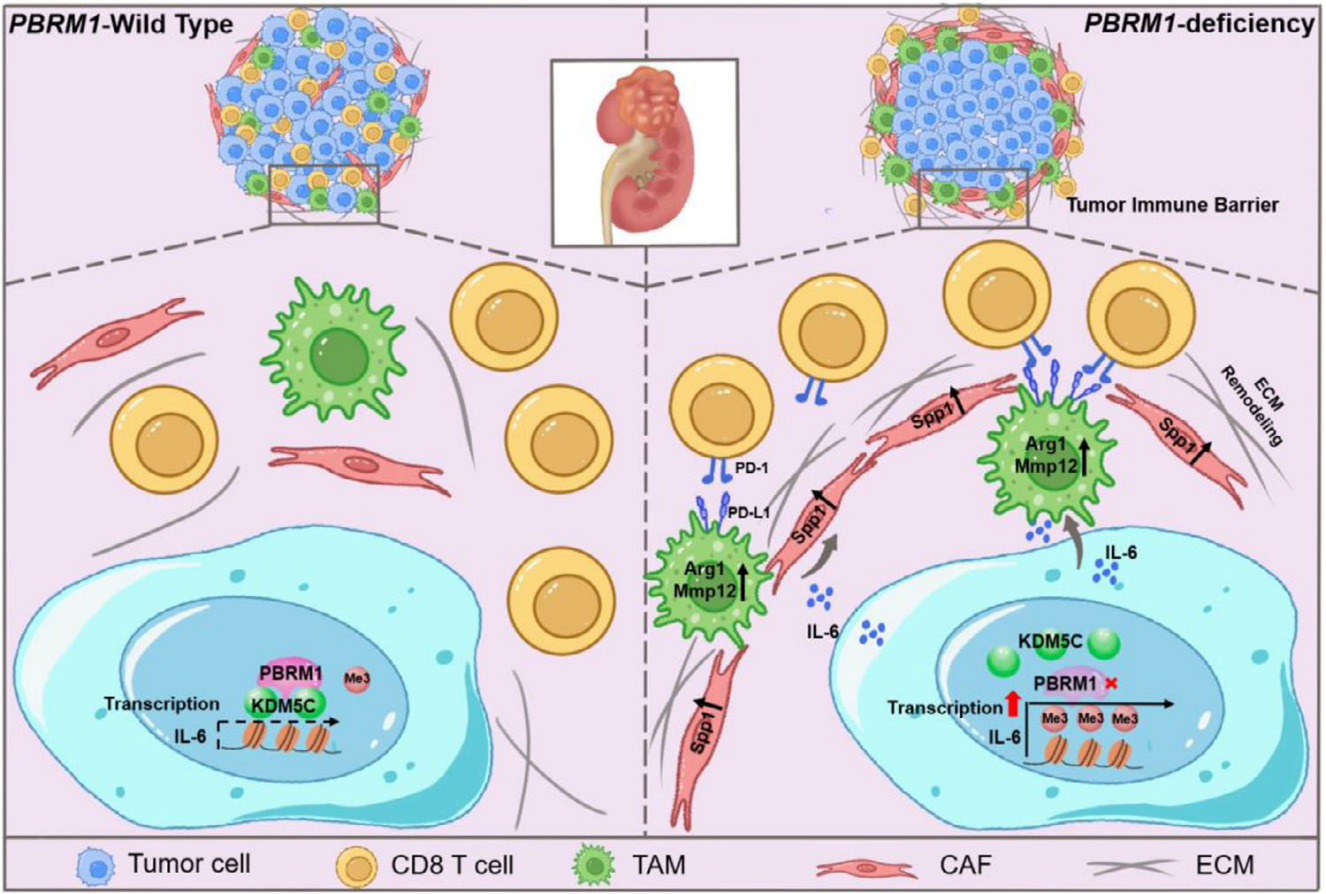
A proposed model for this study.

In summary, our findings illuminate the regulatory role of PBRM1 in shaping the immune landscape of the TME in ccRCC through its interaction with IL‐6. This highlights a crucial signaling axis that could serve as a promising pharmacological target to enhance the sensitivity to ICIs in ccRCC (Figure 8).

## 3 Discussion

Our work introduced an “immune excluded” phenotype in PBRM1‐deficient ccRCC, characterized by the formation of the TIB structure and diminished CD8^+^ T‐cell infiltration, also presented novel insights into the molecular mechanism by which PBRM1 regulates IL‐6 to influence the M2 polarization of macrophages.

*PBRM1* is a common mutated gene in ccRCC, second only to *VHL*. The mutation types of PBRM1 exhibit considerable heterogeneity, including missense mutations, nonsense mutations and frameshift mutations, all of which result in the loss‐of‐function of PBRM1 [47, 48, 49]. PBRM1 is regarded as a tumor suppressor, and in ccRCC, PBRM1 mutations are associated with an unfavorable prognosis. In this study, we developed a genetically modified *Vhl^f/f^* and *Pbrm1^f/f^* mice model, which was first introduced by Gu et al. [50], and strive to be the first to apply it to the field of cancer immunology in PRBM1‐deficient ccRCC. We used the spontaneous tumors to demonstrate a significant enrichment of M2 TAMs, and identified two subgroups, labeled as “Spp1^+^ Mac” and “Retnla^+^ Mac.” Besides, mIHC revealed the substantial colocalization of CD8^+^ T cells and M2 TAMs in the peritumoral region. These data suggest that PBRM1 is involved in regulating the immune system.

Subsequently, mIHC of human PBRM1‐deficient ccRCC specimens clearly illustrated the presence of the TIB structure and the reduction of CD8^+^ T cells within the tumor. This phenomenon was also uncovered in Pbrm1 KO mouse xenograft models. For the details, we found that Mmp12^+^ TAMs contributed to shaping the stroma‐enriched and immunosuppressive TME. Specifically, Mmp12^+^ TAMs can interact with Spp1^+^ CAFs through ligand‐receptor interactions and spatially colocalize with Mmp12^+^ TAMs in the TIB structure to promote the accumulation of ECM components. In addition to MMP12, through the RNA‐seq of M2‐like macrophages, we characterized other two members of MMP family, MMP2 and MMP9, which may have similar effects on fibroblasts and ECM remodeling [51, 52, 53, 54]. Taken together, our work strongly indicated that M2 TAMs regulated ECM remodeling through their interactions with CAFs, thereby promoting the formation of TIB structure and excluding CD8^+^ T cells. It is believed that the TIB structure is an indicative of immunosuppressive TME [55, 56, 57]. Hence, our findings give rise to a critical explanation of why PBRM1 loss has a negative impact on antitumor immunity. Notably, there are controversial labels between spontaneous tumors and orthotopic tumors based on scRNA‐seq analysis. We identified two main reasons for this unique phenomenon. First, one model uses a genetically engineered mouse strain, C57BL/6, while the other utilizes the Balb/C strain. Second, the control group for PBRM1 KO orthotopic tumors consists of PBRM1 WT orthotopic tumors, whereas the control group for spontaneous tumors is healthy kidney tissues. This difference leads to variations in gene expression and marker labeling. We consider that this observed discrepancy is not contradictory but rather reflects the genuine heterogeneity in tumor biology and immune microenvironment simulated by the different models. The genetically engineered mouse mode recapitulates the complete process from tumor initiation and progression to immune editing, resulting in a TME shaped by long‐term, dynamic evolution. The enrichment of Spp1^+^ (associated with matrix remodeling and immunosuppression) and Retnla^+^ (Fizz1, a canonical M2 marker) macrophages strongly suggests a highly immunosuppressive and pro‑fibrotic microenvironment. This is consistent with the stronger CD8^+^ T‑cell functional suppression and enhanced CAF activation observed in this model. In contrast, the orthotopic model involves implanting established tumor cell lines into immunocompetent hosts, leading to a more “acute” process of microenvironment formation. Mmp12^+^ macrophages are closely linked to extracellular matrix degradation, inflammatory responses, and specific chemokine milieus. Their high expression likely reflects the inflammatory state unique to this model‐a condition where rapid tumor growth intersects with host defense reactions. Current understanding indicates that tumor‑associated macrophages (TAMs) do not exist as fixed M1/M2 binary states but rather display a continuous functional spectrum. Spp1, Retnla, and Mmp12 can all be viewed as context‑specific activation markers of M2‑like or immunosuppressive TAMs. Their differential expression likely indicates functional polarization of macrophages adapted to the distinct microenvironmental pressures‐such as cytokine profiles, hypoxia levels, and matrix stiffness‐created by each model. Moreover, multiple studies have demonstrated that while immune‑cell subtype markers may differ between the MC‑38 colon cancer model (C57 background) and the CT‑26 colon cancer model (Balb/c background)‐both commonly used to study colorectal cancer‐the overall immune trends remain consistent across these models [58, 59].

Regarding the question of how M2 TAMs were polarized, our study found that IL‐6 played a crucial role in the background of PBRM1 loss‐of‐function. We confirmed through RNA‐seq, MS analysis of cell supernatants, and ELISA experiments that IL‐6 is significantly upregulated in the PBRM1 KO cancer cells compared to WT. ELISA analysis of patient samples further demonstrated that specimens exhibiting low expression of PBRM1 had markedly elevated levels of IL‐6 compared to those with high PBRM1 expression. Further, by performing epigenomic and gene expression profiling, we reveal the epigenetic mechanism by which PBRM1, as a component of the SWI/SNF complex, regulates IL‐6 expression. We found that loss of PBRM1 leads to increased H3K4me3 peaks at promotors of IL‐6, leading to transcriptional activation.

High IL‐6 expression in ccRCC also indicates an unfavorable prognosis [60, 61]. IL‐6 could activate JAK‐STAT pathway in macrophages and promote immune evasion in a variety of cancers, including hepatocellular carcinoma [62]. KDM5C/JARID1C is a histone demethylase that removes methyl groups from tri‐methylated lysine four on H3K4me3 [63, 64], a histone mark that is tightly linked to actively transcribed genes [65]. KDM5C mutations occur in 3%–7% of ccRCC cases [66]. Notably, KDM5C‐deficient mice mount reduced CD8^+^ T cell due to decreased antigen presentation by cDC1s [67, 68]. This study proved that PBRM1 was able to recruit KDM5C, thereby decreasing H3K4me3 and IL‐6 levels. It is of vital importance that PBRM1‐KDM5C‐IL‐6 axis may have a critical impact on immune regulation in the TME, especially in ccRCC. Since KDM5C belongs to the KDM5 family, it is intriguing to investigate whether other KDM5 family members have the similar function in future explorations.

The genomic and immune microenvironment of ccRCC is characterized by enormous complexity and diversity, highlighting one of the major issues influencing clinical treatment. In this study, we elaborate the molecular mechanism by which PBRM1 loss regulates the tumor immune microenvironment through tuning KDM5C‐IL‐6 axis. This underscores the critical importance of further investigating the pivotal role of IL‐6 in PBRM1‐deficient ccRCC. Notably, the combination of anti‐IL‐6 therapy with anti‐PD‐L1 treatment significantly enhanced the antitumor effects compared to anti‐PD‐L1 monotherapy. Given that the IL‐6 neutralizing antibody (tocilizumab) has already been applied in clinical settings, an in‐depth study of IL‐6 may provide new preclinical evidence for immunotherapy in PBRM1‐deficient ccRCC.

## 4 Experimental Section

### 4.1 Patients and Specimens

We have established a clinical cohort comprising 313 ccRCC patients, the clinical and pathological characteristics of this cohort are presented in Table S1. Tissue samples of ccRCC patients were from the Fourth Affiliated Hospital, Zhejiang University School of Medicine. Informed consent was obtained from all study subjects, and sample collection was approved by the Ethics Committee of the Fourth Affiliated Hospital, Zhejiang University School of Medicine. (K2024182).

### 4.2 Animals

Balb/C mice and NSG mice were purchased from Charles River Laboratories (Beijing, China). *Vhl^f/f^Pbrm1^f/f^Ksp‐Cre* and C57BL/6 mice were purchased from the Shanghai Nanfang Model Organisms Center (Shanghai, China). All animals were kept in specific pathogen‐free conditions. Mice were housed in a standard animal facility, with a 12‐h light/dark cycle, in specific‐pathogen‐free environment. All experiments with mice were conducted in accordance with protocols (ZJU20220502) approved by the Laboratory Animal Welfare and Ethics Review Committee of Zhejiang University. In vivo MAb anti‐mouse PD‐1(BE0146) and InVivoMAb anti‐IL6R antibody (BE0047) were purchased from BioXcell.

### 4.3 Cell Lines

HK2 (CVCL_YE28), 786‐O (CVCL_1051), 769‐P (CVCL_1050), A498 (CVCL_1056) and HEK293T (CVCL_0063) were purchased from American Type Culture Collection (ATCC; Manassas, VA, USA). ACHN (CVCL_1067), Renca (CVCL_2174), Caki‐1 (CVCL_0234) and human monocytic‐leukemia THP‐1 (CVCL_0006), U937 (CVCL_0007) cell lines were purchased from Cell Bank of the Chinese Academy of Sciences (Shanghai, China). 786‐O, 769‐P, Caki‐1, Renca, THP‐1 and U937 cells were cultured in RPMI‐1640 (11875093, Gibco, Grand Island, NY, USA), media supplemented with 100 U/mL Penicillin (V900929, Sigma–Aldrich, St. Louis, MO, USA)/Streptomycin (V900929, Sigma–Aldrich), and 10% heat‐inactivated Fetal bovine serum (10099‐141, Gibco). HK2, A498 and ACHN were maintained in MEM (35271101, Gibco) containing 10% FBS, 1×nonessential amino acids (11140050, Gibco), 100 U/mL Penicillin /Streptomycin. HEK293T was maintained in DMEM (C11995500BT, Gibco) supplemented with 100 U/mL Penicillin /Streptomycin, and 10% FBS. All cells were maintained at 37°C in a humidified incubator with 5% CO_2_. To ensure the absence of mycoplasma contamination, routine screenings were conducted using the MycoAlert Detection Kit (TransGen, FM311‐01).

### 4.4 Antibodies and Reagents

The antibodies and reagents used in this study are listed in Table S10.

### 4.5 Histology

Harvested mouse tissues were fixed in 4% paraformaldehyde (PFA) overnight, embedded in paraffin, and sectioned using a microtome (Leica). Tissue sections were stained with H&E using standard protocols. For IHC analysis, sections were boiled in antigen retrieval solution for 15 min and then brought to room temperature. Subsequently, sections were incubated with specific antibodies, and using the Biotin‐Streptomycin Complex System (Zhongshan Golden Bridge) or a multiplex fluorescent immunohistochemical staining kit (Absin) according to the manufacturer's instructions to visualize the antigen. Images were taken using Digital Slide Scanners (3D HISTECH) or TissueFAXS Spectra Systems (TissueGnostics GmbH).

### 4.6 Fluorescence Signal Quantification

Panoramic multispectral scanning of slides was performed by the Tissue‐FAXS system (TissueFAXS Spectra, TissueGnostics). Then, we imported the data into Strata‐Quest analysis software. We used the spectral library for spectral splitting to obtain a single‐channel fluorescence signal. The DAPI channel was used to identify the effective nucleus. Using the effective nucleus as the core, we set the distance radius according to the staining of each protein channel and found the protein fluorescent staining signal. Then, we set the threshold according to the staining situation of each channel, divided the positive cell population, and counted the positive cells. We also counted the number and intensity of double‐positive cells. We used the mean intensity to multiplicate positive cells and divided them by the total cell number to generate the gene expression mean.

### 4.7 Isolation of CAFs, Macrophages and Tumor Cells from RCC Tissues

Briefly, primary RCC tissues were obtained from the operating room during nephrectomy. Tissues were rinsed in HBSS (Gibco) until there was no visible blood then sliced into approximately 30 mm^3^ pieces of tissue and digested for 30 min at 37°C with agitation in a digestion solution containing 1 mg/mL collagenase (Sigma–Aldrich) and 50 µg/mL DNase (Sigma–Aldrich) in RPMI (Gibco). Sample dissociation solutions were filtered by a 40‐µm cell strainer and were neutralized with complete medium and centrifuged at 300 g for 5 min. Then, the mixed cells were sorted to tumor cells, macrophages, and CAFs using MACS(Magnetic Activated Cell Sorting (Miltenyi Biotec).

### 4.8 Patient‐Derived Organoids (PDO)

ccRCC tumor tissues were washed and digested at 37°C for 50 min. Dissociated tissues were spun down for 10 min and resuspended in cell medium. Dissociated cell clusters were centrifuged again. Cell clusters were resuspended in BME buffer on ice and plated as a 300 µL drop within a 12 mm, 0.4 µm inner Transwell chamber. The drop was solidified by a 30‐min incubation at 37°C and 5% CO_2_ with 1 mL of renal cancer organoid medium. The growth state of organoids was monitored on day 1, 3, 10. Hematoxylin & eosin (H&E) staining was performed on tumor organoids to reveal cancer nuclear division pattern and atypia.

### 4.9 Isolation of Mouse Lymphocytes and Flow Cytometry

Mouse spleen was harvested and passed through 100 mm filters using flow cytometry buffer (FACS buffer; 1x D‐PBS, 2% FCS). Tumors were resected and enzymatically digested with 1 mg/mL Collagenase D (Roche) and 50 ng/mL DNase1 for 30 min at 37°C while under constant agitation. All samples were treated with ACK lysis buffer (0.15 M NH4Cl, 0.01 M KHCO3, 0.1 mm NaEDTA) for 5 min at room temperature to lyse red blood cells. Single cell suspensions were washed and resuspended with FACS buffer. Cell suspensions were subjected to Fc receptor blocking, and stained with conjugated antibodies for 30 min at 4°C in the dark, washed twice in PBS, and then tested. For intracellular staining, surface antigens were stained first, followed by fixation and permeabilization with Foxp3/transcription factor staining buffer. BD Celesta flow cytometry was used for detection, and data were analyzed with Flowjo V10.

### 4.10 BUN and Creatinine Measurements

Blood was collected in heparinized tubes before mice were sacrificed and plasma was obtained by spinning down samples at 3000 rpm for 10 min within 30 min of collection. Plasma samples were kept in a −20°C freezer until submission to the mouse metabolic phenotyping core for measurement of BUN and creatinine levels by Beckman Coulter AU680 analyzer.

### 4.11 Lentiviral Transduction and Screening for Stable Cell Lines

PBRM1 or KDM5C were knocked out using CRISPR‐Cas9‐mediated gene editing. Phosphorylated and annealed sense and antisense oligos were ligated into BsmBI digested vectors. sgRNAs were cloned into the BsmBI digested LentiCRISPR v2 backbone (human PBRM1 sgRNA: 5’‐TCAGCGGGGACTTTGATGAT‐3’, 5’‐ AGACTATAAGGATGAACAGG‐3’; mouse Pbrm1 sgRNA: 5’‐TGCCTACACCAGGCCCAAGC‐3’, 5’‐TAACACCATCC GAGACTATA‐3’; human KDM5C sgRNA: 5’‐CTGCCGGATGTGTTCTCGAG‐3’, 5’‐GTCTGCCGGATGTGTTCTCG‐3’). The KDM5C overexpression lentiviral vectors, lentiviral Flag‐PBRM1 and mutants were generated into pLNCX‐GFP lentiviral vector. The PBRM1‐sg, KDM5C‐sg, KDM5C‐OE, Flag‐PBRM1 and mutants were generated by lentiviral transduction according to the manufacturer's protocol. The cells with stable expression were identified by screening with culture medium supplemented with puromycin (Yeasen) at a final concentration of 5 µg/mL after 2 weeks.

### 4.12 RNA Isolation and qRT‐PCR

Total RNA was extracted from the indicated cells and frozen tissue specimens using TRIzol reagent (Invitrogen, USA) and reverse transcribed into cDNA using a Prime Script RT reagent kit (Takara, Japan) according to the manufacturers'instructions. For qPCR, a reaction system containing cDNA, primers, and SYBR Green Master Mix was run on an ABI Prism 7500 Sequence Detection System (Applied Biosystems). The expression of the target gene was normalized to that of the housekeeping gene GAPDH and quantified using the ∆∆Ct method.

### 4.13 Cell Proliferation and Viability Assays

Cell proliferation was measured using an CCK8 cell proliferation kit (Roche). Briefly, 1 × 10^4^ cells were plated in triplicate in 96‐well plates. 72 h later, 10 µl of CCK8 labeling reagent was added to each well and then incubated for 3 h at 37°C, absorbance was measured at 590 nm on a plate reader (Tecan Safire).

### 4.14 Western Blot Assay and Immunoprecipitation (IP)

For western blotting, proteins were extracted by lysing cultured cells in RIPA buffer containing protease inhibitors and quantified using a BCA assay kit. Later, heat denatured proteins mixed with 1 × loading buffer were separated by SDS–PAGE and then transferred onto PVDF membranes (Millipore, USA). Finally, the blots were exposed with enhanced chemiluminescence reagents using ImageQuant LAS 4000 after incubation with the indicated primary antibodies followed by HRP‐conjugated secondary antibody.

For IPs, cell lysates were incubated with the indicated antibodies at 4°C overnight on a vertical rotator and then rotated vertically for 30 min at room temperature in the presence of protein A/G beads. Afterward, the beads were washed five times with precooled lysis buffer and mixed with SDS‐PAGE loading buffer to elute proteins for immunoblotting.

### 4.15 ELISA

The tissue supernatant or cell supernatant was collected after centrifugation, and the concentration of cytokines was measured using ELISA kits according to the manufacturer's instructions; the ELISA kits used were as follows: IL‐6 ELISA kit (Dakewe), Granym B ELISA kit (Dakewe), Perforin ELISA kit (Dakewe), and IFN‐g ELISA kit (Dakewe).

### 4.16 BMDM Isolation

Bone marrow extraction was performed as previously described [69]. Cells were plated in RPMI with 10% FBS, 1% Pen/Strep and 50 ng/mL mouse macrophage colony‐stimulating factor (BioLegend). On day 7, BMDMs were polarized using cytokines and cancer cells. For the coculture experiments, PBRM1 WT or PBRM1 KO cancer cells were seeded at 1 × 10^5^ cells/mL into a 0.4 µm insert a day before the polarization experiment. The next day, inserts containing cancer cells were transferred to a six‐well plate seeded with M0 polarized BMDMs. Cells were harvested 48 h after polarization for further analyses. For FACS analyses, 1.5 × 10^5^ cancer cells in total were plated directly onto polarized BMDMs for 48 h and then collected and stained as described in the flow cytometry section.

### 4.17 ChIP qRT‐PCR Assays

ChIP qRT‐PCR assays were performed using the EpiQuik Chromatin Immunoprecipitation Kit (Epigentek, Farmingdale, NY, USA) following the protocol supplied by the manufacturer. For the ChIP assays, 1 × 10^6^ cells were fixed in 1% formaldehyde for 10 min. Lysed samples were sonicated to fragment the DNA. The DNA‐bound proteins were immunoprecipitated with antibodies directed against H3K4me3 (Epigentek). The collected DNA and input samples were analyzed for associated DNA fragments using quantitative qRT‐PCR.

### 4.18 scRNA‐Seq

The fresh tissues were stored in the sCelLiveTM Tissue Preservation Solution (Singleron) on ice after the surgery within 30 min. The specimens were washed with Hanks Balanced Salt Solution (HBSS) for three times, minced into small pieces, and then digested with 3 mL sCelLiveTM Tissue Dissociation Solution (Singleron) by Singleron PythoN Tissue Dissociation System at 37°C for 15 min. The cell suspension was collected and filtered through a 40‐micron sterile strainer. Afterward, the GEXSCOPE red blood cell lysis buffer (RCLB, Singleron) was added, and the mixture [Cell: RCLB = 1:2 (volume ratio)] was incubated at room temperature for 5–8 min to remove red blood cells. The mixture was then centrifuged at 300 × g 4°C for 5 mins to remove supernatant and suspended softly with PBS. Finally, the samples were stained with Trypan Blue and the cell viability was evaluated microscopically. Single‐cell suspensions (2 × 10^5^ cells/mL) with PBS (HyClone) were loaded onto microwell chip using the Singleron Matrix Single Cell Processing System. Barcoding Beads are subsequently collected from the microwell chip, followed by reverse transcription of the mRNA captured by the Barcoding Beads and to obtain cDNA, and PCR amplification. The amplified cDNA is then fragmented and ligated with sequencing adapters. The scRNA‐seq libraries were constructed according to the protocol of the GEXSCOPE Single Cell RNA Library Kits (Singleron) (Ref). Individual libraries were diluted to 4 nm, pooled, and sequenced on Illumina NovaSeq X Plus with 150 bp paired end reads.

### 4.19 scRNA‐Seq Analysis

Quality control, dimensionality reduction, and clustering were performed using Scanpy (v1.8.1) in Python 3.7 [70]. Data filtering criteria included: excluding cells with fewer than 200 genes or top 2% of gene counts, top 2% UMI counts, and cells with over 50% mitochondrial content. Post‐filtering, 24 370 cells with an average of 1140 genes and 3178 UMIs per cell were retained. Raw count matrices were normalized by total counts per cell and log‐transformed. Variable genes (top 2000) were selected, and principal component analysis (PCA) was conducted on scaled data, retaining 20 components. Clustering was done with the Louvain algorithm (resolution = 1.2), and clusters were visualized with UMAP. DEGs were identified using the scanpy.tl.rank_genes_groups() function with a Wilcoxon rank‐sum test. Genes expressed in over 10% of cells in either group, with a log2 fold change >1, were designated as DEGs, and Benjamini–Hochberg correction was applied with an adjusted *p*‐value threshold of 0.05. Pathway enrichment analysis was conducted using “clusterProfiler” (v4.0.0) in R. Pathways with adjusted p‐values <0.05 were deemed significantly enriched, and key pathways were visualized with bar plots. Cell type annotation was based on canonical markers from SynEcoSys (Singleron Biotechnology), incorporating markers from databases like CellMakerDB and PanglaoDB. CellChat (v1.6.1) was employed to study cell‐cell communication, using ligand‐receptor data and generating visualizations [71]. CellPhoneDB (v4.0.0) was also used to predict interactions, with significant pairs (*p* < 0.05) visualized as heatmaps and dot plots [72]. Cell differentiation trajectories were constructed using Monocle2 (v2.22.0), with highly variable genes selected for dimension reduction via DDRTree [73]. Trajectories were visualized with plot_cell_trajectory(). Transcription factor network was constructed with pySCENIC (v1.1.2) [74]. InferCNV was applied to detect CNAs, with non‐malignant T cells as a baseline for comparison with malignant epithelial cells. CNV scores per cell were calculated and visualized with heatmaps and clonality trees using UPhyloplot2 [75].

### 4.20 Bulk RNA‐Seq

poly‐T oligo‐attached magnetic beads were used to purify messenger RNA from total RNA. Following fragmentation, the first‐strand cDNA was synthesized using random hexamer primers, while the second strand was synthesized with dUTP. The directional library was then prepared through a series of steps including end repair, A‐tailing, adapter ligation, size selection, USER enzyme digestion, amplification, and purification. Each sample was processed with three biological replicates, and 20 million reads were generated per replicate on Illumina NovaSeq 6000 sequencer.

### 4.21 Bulk RNA‐Seq Analysis

Sequencing quality was assessed with FastQC (v0.12.1) (https://github.com/s‐andrews/FastQC), and read quality was controlled using Trim Galore (v0.6.7) (https://github.com/FelixKrueger/TrimGalore). The reads were aligned to the human reference genome (hg38) using STAR (v2.7.11) [76], and gene expression was quantified with HTSeq‐count (v2.0.4) [77]. Downstream statistical analyses were conducted using RStudio and differential gene expression analysis was performed using DESeq2 (v1.38.3) (https://bioconductor.org/packages/release/bioc/html/DESeq2.html).

### 4.22 Bulk ATAC‐Seq and ChIP‐Seq Analysis

Sequencing quality was assessed with FastQC (v0.12.1) (https://github.com/s‐andrews/FastQC), and read quality was controlled using Trim Galore (v0.6.7) (https://github.com/FelixKrueger/TrimGalore). The reads were aligned to the human reference genome (hg38) using Bowtie 2 (v2.5.1) [78], and duplicates were marked and removed with SAMtools (v1.13) [79]. For ATAC‐seq, peak calling was performed using MACS2 (v2.2.9.1) with the parameters: ‐f BEDPE, ‐ghs, –shift ‐100, –extsize 200, ‐q 1e‐2, –nomodel, ‐B, and ‐SPMR [80]. For ChIP‐seq, peak calling was done with the parameters: ‐f BAMPE, ‐ghs, –nomodel, ‐B, and ‐q 1e‐2. BigWig tracks were generated using deepTools bamCoverage (v3.5.1) with RPGC normalization [81]. Differential analysis between KO and WT samples was conducted using BEDTools (v2.30.0) [82]. Quality of ATAC‐seq and ChIP‐seq data was evaluated according to the ENCODE ATAC‐seq and histone ChIP‐seq data standards. Genome tracks were visualized using IGV (v2.14.1) [83]. A public access version is also available: PMC3346182.

### 4.23 Survival Analysis

Kaplan‐Meier survival analysis was used to assess the relationship of the signature scores and PBRM1 expression with OS. We applied the large‐sample Chi‐square test (log‐rank test) to determine the associations between predictor variables and to obtain adjusted hazard‐ratios. These analyses were performed with the R package “survival.”

### 4.24 Quantification and Statistical Analysis

All statistical analyses described above were performed either with R, or with Prism 9 (GraphPad Software).

## Author Contributions

C.Z., G.Z. and X.C. conceived and conducted the project. C.Z. supervised the project. W.X., H.W., Y.D., Z.Y. and Y.Z. performed the experiments, analyzed data, and wrote the paper. Z.X., L.R., Q.L. and Z.C. contributed to the cell culture experiments and mouse models. Y.Z. collected tissue samples and information from patients. J.Y. and D.M. contributed to the imaging analysis and interpreted the data.

## Funding

This work was supported by Key project of National Natural Science Foundation of China (no. 82330094), National Natural Science Foundation of China (no.32370749), Key project of provincial Natural Science Foundation (no. LZ22H160008), and the Natural Science Foundation of Zhejiang Province, Exploration Project/Exploration Public welfare/Medical health (no. LTGY24H160020).

## Conflicts of Interest

The authors declare no conflicts of interest.

## Supporting information




**Supporting File 1**: advs73755‐sup‐0001‐SuppMat.docx.

**Supporting File 2**: advs73755‐sup‐0002‐Table S1.xlsx.

**Supporting File 3**: advs73755‐sup‐0003‐TableS2.xlsx.

**Supporting File 4**: advs73755‐sup‐0004‐TableS3.xlsx.

**Supporting File 5**: advs73755‐sup‐0005‐TableS4.xlsx.

**Supporting File 6**: advs73755‐sup‐0006‐TableS5.xlsx.

**Supporting File 7**: advs73755‐sup‐0007‐TableS6.xlsx.

**Supporting File 8**: advs73755‐sup‐0008‐TableS7.xlsx.

**Supporting File 9**: advs73755‐sup‐0009‐TableS8.xls.

**Supporting File 10**: advs73755‐sup‐0010‐TableS9.xls.

**Supporting File 11**: advs73755‐sup‐0011‐TableS10.docx.

## Data Availability

The raw sequence data reported in this paper have been deposited in the Genome Sequence Archive [84] in National Genomics Data Center [85], China National Center for Bioinformation/Beijing Institute of Genomics, Chinese Academy of Sciences (GSA‐Human: HRA015241 and HRA015272; GSA: CRA034528) that are publicly accessible at https://ngdc.cncb.ac.cn/gsa‐human. The bulk ATAC‐seq data was sourced from the NCBI Gene Expression Omnibus (GEO) under accession GSE102807, and the histone modification ChIP‐seq data were obtained from GEO under accession GSE152681. The scRNA‐seq data was sourced from GEO under accession GSE227898 and NCBI Sequence Read Archive (SRA) under accession PRJNA768891. All data are available in the main text or the supplementary materials.

## References

[1] Y. Senbabaoglu , R. S. Gejman , A. G. Winer , et al., “Tumor Immune Microenvironment Characterization in Clear Cell Renal Cell Carcinoma Identifies Prognostic and Immunotherapeutically Relevant Messenger RNA Signatures,” Genome Biology 17, no. 1 (2016): 231, 10.1186/s13059-016-1092-z. 27855702 PMC5114739

[2] M. S. Rooney , S. A. Shukla , C. J. Wu , G. Getz , and N. Hacohen , “Molecular and Genetic Properties of Tumors Associated with Local Immune Cytolytic Activity,” Cell 160, no. 1‐2 (2015): 48–61, 10.1016/j.cell.2014.12.033. 25594174 PMC4856474

[3] L. Bukavina , K. Bensalah , F. Bray , et al., “Epidemiology of Renal Cell Carcinoma: 2022 Update,” European Urology 82, no. 5 (2022): 529–542, 10.1016/j.eururo.2022.08.019. 36100483

[4] D. A. Braun , Y. Ishii , A. M. Walsh , et al., “Clinical Validation of PBRM1 Alterations as a Marker of Immune Checkpoint Inhibitor Response in Renal Cell Carcinoma,” JAMA Oncology 5, no. 11 (2019): 1631–1633, 10.1001/jamaoncol.2019.3158. 31486842 PMC6735411

[5] D. A. Braun , Y. Hou , Z. Bakouny , et al., “Interplay of Somatic Alterations and Immune Infiltration Modulates Response to PD‐1 Blockade in Advanced Clear Cell Renal Cell Carcinoma,” Nature Medicine 26, no. 6 (2020): 909–918, 10.1038/s41591-020-0839-y. PMC7499153 32472114

[6] R. J. Motzer , B. Escudier , D. F. McDermott , et al., “Nivolumab Versus Everolimus in Advanced Renal‐Cell Carcinoma,” New England Journal of Medicine 373, no. 19 (2015): 1803–1813, 10.1056/NEJMoa1510665. 26406148 PMC5719487

[7] R. J. Motzer , E. Jonasch , N. Agarwal , et al., “NCCN Guidelines® Insights: Kidney Cancer, Version 2.2024,” Journal of the National Comprehensive Cancer Network 22, no. 1 (2024): 4–16, 10.6004/jnccn.2024.0008. 38394781

[8] M. Yarchoan , A. Hopkins , and E. M. Jaffee , “Tumor Mutational Burden and Response Rate to PD‐1 Inhibition,” New England Journal of Medicine 377, no. 25 (2017): 2500–2501, 10.1056/NEJMc1713444. 29262275 PMC6549688

[9] B. I. Rini , E. R. Plimack , V. Stus , et al., “Pembrolizumab Plus Axitinib Versus Sunitinib for Advanced Renal‐Cell Carcinoma,” New England Journal of Medicine 380, no. 12 (2019): 1116–1127, 10.1056/NEJMoa1816714. 30779529

[10] D. F. McDermott , M. A. Huseni , M. B. Atkins , et al., “Clinical Activity and Molecular Correlates of Response to Atezolizumab Alone or in Combination with Bevacizumab Versus Sunitinib in Renal Cell Carcinoma,” Nature Medicine 24, no. 6 (2018): 749–757, 10.1038/s41591-018-0053-3. PMC6721896 29867230

[11] R. M. Saliby , C. Labaki , T. R. Jammihal , et al., “Impact of Renal Cell Carcinoma Molecular Subtypes on Immunotherapy and Targeted Therapy Outcomes,” Cancer Cell 42, no. 5 (2024): 732–735, 10.1016/j.ccell.2024.03.002. 38579722 PMC11130783

[12] P. Sharma , S. Hu‐Lieskovan , J. A. Wargo , and A. Ribas , “Primary, Adaptive, and Acquired Resistance to Cancer Immunotherapy,” Cell 168, no. 4 (2017): 707–723, 10.1016/j.cell.2017.01.017. 28187290 PMC5391692

[13] J. M. Pitt , M. Vetizou , R. Daillere , et al., “Resistance Mechanisms to Immune‐Checkpoint Blockade in Cancer: Tumor‐Intrinsic and ‐Extrinsic Factors,” Immunity 44, no. 6 (2016): 1255–1269, 10.1016/j.immuni.2016.06.001. 27332730

[14] P. S. Hegde and D. S. Chen , “Top 10 Challenges in Cancer Immunotherapy,” Immunity 52, no. 1 (2020): 17–35, 10.1016/j.immuni.2019.12.011. 31940268

[15] J. A. Trujillo , R. F. Sweis , R. Bao , and J. J. Luke , “T Cell–Inflamed Versus Non‐T Cell–Inflamed Tumors: A Conceptual Framework for Cancer Immunotherapy Drug Development and Combination Therapy Selection,” Cancer Immunology Research 6, no. 9 (2018): 990–1000, 10.1158/2326-6066.CIR-18-0277. 30181337 PMC6145135

[16] P. C. Tumeh , C. L. Harview , J. H. Yearley , et al., “PD‐1 Blockade Induces Responses by Inhibiting Adaptive Immune Resistance,” Nature 515, no. 7528 (2014): 568–571, 10.1038/nature13954. 25428505 PMC4246418

[17] The Cancer Genome Atlas Research , “Comprehensive Molecular Characterization of Clear Cell Renal Cell Carcinoma,” Nature 499, no. 7456 (2013): 43–49, 10.1038/nature12222. 23792563 PMC3771322

[18] I. Lyskjaer , L. Iisager , C. T. Axelsen , T. K. Nielsen , L. Dyrskjot , and N. Fristrup , “Management of Renal Cell Carcinoma: Promising Biomarkers and the Challenges to Reach the Clinic,” Clinical Cancer Research 30, no. 4 (2024): 663–672, 10.1158/1078-0432.CCR-23-1892. 37874628 PMC10870122

[19] S. Turajlic , H. Xu , K. Litchfield , et al., “Tracking Cancer Evolution Reveals Constrained Routes to Metastases: TRACERx Renal,” Cell 173, no. 3 (2018): 581–594, 10.1016/j.cell.2018.03.057. 29656895 PMC5938365

[20] C. J. Ricketts , A. A. De Cubas , H. Fan , et al., “The Cancer Genome Atlas Comprehensive Molecular Characterization of Renal Cell Carcinoma,” Cell Reports 23, no. 1 (2018): 313–326, 10.1016/j.celrep.2018.03.075. 29617669 PMC6075733

[21] Z. Bakouny , D. A. Braun , S. A. Shukla , et al., “Integrative Molecular Characterization of Sarcomatoid and Rhabdoid Renal Cell Carcinoma,” Nature Communications 12, no. 1 (2021): 808, 10.1038/s41467-021-21068-9. PMC7865061 33547292

[22] P. M. Brownlee , A. L. Chambers , R. Cloney , A. Bianchi , and J. A. Downs , “BAF180 Promotes Cohesion and Prevents Genome Instability and Aneuploidy,” Cell Reports 6, no. 6 (2014): 973–981, 10.1016/j.celrep.2014.02.012. 24613357 PMC3988838

[23] R. M. Chabanon , D. Morel , T. Eychenne , et al., “PBRM1 Deficiency Confers Synthetic Lethality to DNA Repair Inhibitors in Cancer,” Cancer Research 81, no. 11 (2021): 2888–2902, 10.1158/0008-5472.CAN-21-0628. 33888468

[24] D. Miao , C. A. Margolis , W. Gao , et al., “Genomic Correlates of Response to Immune Checkpoint Therapies in Clear Cell Renal Cell Carcinoma,” Science 359, no. 6377 (2018): 801–806, 10.1126/science.aan5951. 29301960 PMC6035749

[25] N. Dizman , P. G. Bergerot , C. D. Bergerot , J. Hsu , and S. K. Pal , “Duration of treatment (DOT) with targeted therapies (TT) or immunotherapy (IO) in PBRM1 mutated metastatic renal cell carcinoma (mRCC),” Journal of Clinical Oncology 37 (2019): 622.

[26] A. A. Hakimi , Y. Ged , J. R. Flynn , et al., “The impact of PBRM1 mutations on overall survival in greater than 2,100 patients treated with immune checkpoint blockade ICB,” Journal of Clinical Oncology 37, no. 7 (2019): 666.

[27] The Cancer Genome Atlas Network , “Genomic Classification of Cutaneous Melanoma,” Cell 161, no. 7 (2015): 1681–1696, 10.1016/j.cell.2015.05.044. 26091043 PMC4580370

[28] V. Thorsson , D. L. Gibbs , S. D. Brown , et al., “The Immune Landscape of Cancer,” Immunity 48, no. 4 (2018): 812–830, 10.1016/j.immuni.2018.03.023. 29628290 PMC5982584

[29] S. Chevrier , J. H. Levine , V. R. T. Zanotelli , et al., “An Immune Atlas of Clear Cell Renal Cell Carcinoma,” Cell 169, no. 4 (2017): 736–749, 10.1016/j.cell.2017.04.016. 28475899 PMC5422211

[30] T. Wang , R. Lu , P. Kapur , et al., “An Empirical Approach Leveraging Tumorgrafts to Dissect the Tumor Microenvironment in Renal Cell Carcinoma Identifies Missing Link to Prognostic Inflammatory Factors,” Cancer Discovery 8, no. 9 (2018): 1142–1155, 10.1158/2159-8290.CD-17-1246. 29884728 PMC6125163

[31] E. M. Genega , M. Ghebremichael , R. Najarian , et al., “Carbonic Anhydrase IX Expression in Renal Neoplasms,” American Journal of Clinical Pathology 134, no. 6 (2010): 873–879, 10.1309/AJCPPPR57HNJMSLZ. 21088149 PMC3778911

[32] C. C. Wykoff , N. J. Beasley , P. H. Watson , et al., “Hypoxia‐Inducible Expression of Tumor‐Associated Carbonic Anhydrases,” Cancer Research 60, no. 24 (2000): 7075–7083. 11156414

[33] X. Zhang , Y. Lan , J. Xu , et al., “CellMarker: A Manually Curated Resource of Cell Markers in Human and Mouse,” Nucleic Acids Research 47, no. D1 (2019): D721–D728, 10.1093/nar/gky900. 30289549 PMC6323899

[34] D. J. Clark , S. M. Dhanasekaran , F. Petralia , et al., “Integrated Proteogenomic Characterization of Clear Cell Renal Cell Carcinoma,” Cell 179, no. 4 (2019): 964–983, 10.1016/j.cell.2019.10.007. 31675502 PMC7331093

[35] H. Li , J. Chen , Z. Li , et al., “S100A5 Attenuates Efficiency of Anti‐PD‐L1/PD‐1 Immunotherapy by Inhibiting CD8 + T Cell‐Mediated Anti‐Cancer Immunity in Bladder Carcinoma,” Advanced Science 10, no. 25 (2023): 2300110, 10.1002/advs.202300110. 37414584 PMC10477882

[36] A. Harrod , K. A. Lane , and J. A. Downs , “The Role of the SWI/SNF Chromatin Remodelling Complex in the Response to DNA Double Strand Breaks,” DNA Repair 93 (2020): 102919, 10.1016/j.dnarep.2020.102919. 33087260

[37] A. P. Bracken , G. L. Brien , and C. P. Verrijzer , “Dangerous Liaisons: Interplay Between SWI/SNF, NuRD, and Polycomb in Chromatin Regulation and Cancer,” Genes & Development 33, no. 15‐16 (2019): 936–959, 10.1101/gad.326066.119. 31123059 PMC6672049

[38] A. Bayona‐Feliu , S. Barroso , S. Munoz , and A. Aguilera , “The SWI/SNF Chromatin Remodeling Complex Helps Resolve R‐Loop‐Mediated Transcription–Replication Conflicts,” Nature Genetics 53, no. 7 (2021): 1050–1063, 10.1038/s41588-021-00867-2. 33986538

[39] X. Niu , T. Zhang , L. Liao , et al., “The Von Hippel–Lindau Tumor Suppressor Protein Regulates Gene Expression and Tumor Growth Through Histone Demethylase JARID1C,” Oncogene 31, no. 6 (2012): 776–786, 10.1038/onc.2011.266. 21725364 PMC4238297

[40] L. P. Blair , J. Cao , M. R. Zou , J. Sayegh , and Q. Yan , “Epigenetic Regulation by Lysine Demethylase 5 (KDM5) Enzymes in Cancer,” Cancers 3, no. 1 (2011): 1383–1404, 10.3390/cancers3011383. 21544224 PMC3085456

[41] M. Karki , R. K. Jangid , R. Anish , et al., “A Cytoskeletal Function for PBRM1 Reading Methylated Microtubules,” Science Advances 7, no. 14 (2021): abf2866, 10.1126/sciadv.abf2866. PMC11059954 33811077

[42] N. Yang and R. M. Xu , “Structure and Function of the BAH Domain in Chromatin Biology,” Critical Reviews in Biochemistry and Molecular Biology 48, no. 3 (2013): 211–221, 10.3109/10409238.2012.742035. 23181513

[43] M. Stros , D. Launholt , and K. D. Grasser , “The HMG‐Box: A Versatile Protein Domain Occurring in a Wide Variety of DNA‐Binding Proteins,” Cellular and Molecular Life Sciences 64, no. 19‐20 (2007): 2590, 10.1007/s00018-007-7162-3. 17599239 PMC11136187

[44] L. M. McLane , M. S. Abdel‐Hakeem , and E. J. Wherry , “CD8 T Cell Exhaustion During Chronic Viral Infection and Cancer,” Annual Review of Immunology 37 (2019): 457–495, 10.1146/annurev-immunol-041015-055318. 30676822

[45] P. Sharma and J. P. Allison , “The Future of Immune Checkpoint Therapy,” Science 348, no. 6230 (2015): 56–61, 10.1126/science.aaa8172. 25838373

[46] A. C. Huang , M. A. Postow , R. J. Orlowski , et al., “T‐Cell Invigoration to Tumour Burden Ratio Associated with Anti‐PD‐1 Response,” Nature 545, no. 7652 (2017): 60–65, 10.1038/nature22079. 28397821 PMC5554367

[47] W. Gao , W. Li , T. Xiao , X. S. Liu , and W. G. Kaelin Jr. , “Inactivation of the PBRM1 Tumor Suppressor Gene Amplifies the HIF‐Response in VHL −/− Clear Cell Renal Carcinoma,” Proceedings of the National Academy of Sciences 114, no. 5 (2017): 1027–1032, 10.1073/pnas.1619726114. PMC5293026 28082722

[48] A. M. Nargund , C. G. Pham , Y. Dong , et al., “The SWI/SNF Protein PBRM1 Restrains VHL‐Loss‐Driven Clear Cell Renal Cell Carcinoma,” Cell Reports 18, no. 12 (2017): 2893–2906, 10.1016/j.celrep.2017.02.074. 28329682 PMC5431084

[49] I. Varela , P. Tarpey , K. Raine , et al., “Exome Sequencing Identifies Frequent Mutation of the SWI/SNF Complex Gene PBRM1 in Renal Carcinoma,” Nature 469, no. 7331 (2011): 539–542, 10.1038/nature09639. 21248752 PMC3030920

[50] Y. F. Gu , S. Cohn , A. Christie , et al., “Modeling Renal Cell Carcinoma in Mice: Bap1 and Pbrm1 Inactivation Drive Tumor Grade,” Cancer Discovery 7, no. 8 (2017): 900–917, 10.1158/2159-8290.CD-17-0292. 28473526 PMC5540776

[51] M. Aristorena , E. Gallardo‐Vara , M. Vicen , et al., “MMP‐12, Secreted by Pro‐Inflammatory Macrophages, Targets Endoglin in Human Macrophages and Endothelial Cells,” International Journal of Molecular Sciences 20, no. 12 (2019): 3107, 10.3390/ijms20123107. 31242676 PMC6627183

[52] N. Cui , M. Hu , and R. A. Khalil , “Chapter One ‐ Biochemical and Biological Attributes of Matrix Metalloproteinases,” Progress in Molecular Biology and Translational Science 147 (2017): 1–73, 10.1016/bs.pmbts.2017.02.005. 28413025 PMC5430303

[53] C. J. Philp , I. Siebeke , D. Clements , et al., “Extracellular Matrix Cross‐Linking Enhances Fibroblast Growth and Protects Against Matrix Proteolysis in Lung Fibrosis,” American Journal of Respiratory Cell and Molecular Biology 58, no. 5 (2018): 594, 10.1165/rcmb.2016-0379OC. 29053339

[54] H. Huang , Z. Wang , Y. Zhang , et al., “Mesothelial Cell‐Derived Antigen‐Presenting Cancer‐Associated Fibroblasts Induce Expansion of Regulatory T Cells in Pancreatic Cancer,” Cancer Cell 40, no. 6 (2022): 656–673, 10.1016/j.ccell.2022.04.011. 35523176 PMC9197998

[55] Y. Liu , Z. Xun , K. Ma , et al., “Identification of a Tumour Immune Barrier in the HCC Microenvironment that Determines the Efficacy of Immunotherapy,” Journal of Hepatology 78, no. 4 (2023): 770–782, 10.1016/j.jhep.2023.01.011. 36708811

[56] S. P. Fortis , M. Sofopoulos , N. N. Sotiriadou , et al., “Differential Intratumoral Distributions of CD8 and CD163 Immune Cells as Prognostic Biomarkers in Breast Cancer,” Journal for ImmunoTherapy of Cancer 5 (2017): 39, 10.1186/s40425-017-0240-7. 28428887 PMC5395775

[57] C. Oelkrug and J. M. Ramage , “Enhancement of T Cell Recruitment and Infiltration into Tumours,” Clinical and Experimental Immunology 178, no. 1 (2014): 1–8, 10.1111/cei.12382. PMC4360188 24828133

[58] Z. Y. Wu , Q. W. Wu , Y. Han , et al., “Alistipes Finegoldii Augments the Efficacy of Immunotherapy Against Solid Tumors,” Cancer Cell 43, no. 9 (2025): 1714–1730, 10.1016/j.ccell.2025.07.002. 40712567

[59] G. Deng , L. Zhou , B. Wang , et al., “Targeting Cathepsin B by Cycloastragenol Enhances Antitumor Immunity of CD8 T Cells via Inhibiting MHC‐I Degradation,” Journal for ImmunoTherapy of Cancer 10, no. 10 (2022): 004874, 10.1136/jitc-2022-004874. PMC9621195 36307151

[60] S. A. Jones and B. J. Jenkins , “Recent Insights into Targeting the IL‐6 Cytokine Family in Inflammatory Diseases and Cancer,” Nature Reviews Immunology 18, no. 12 (2018): 773–789, 10.1038/s41577-018-0066-7. 30254251

[61] D. E. Johnson , R. A. O'Keefe , and J. R. Grandis , “Targeting the IL‐6/JAK/STAT3 Signalling Axis in Cancer,” Nature Reviews Clinical Oncology 15, no. 4 (2018): 234–248, 10.1038/nrclinonc.2018.8. PMC5858971 29405201

[62] C. M. Wunderlich , P. J. Ackermann , A. L. Ostermann , et al., “Obesity Exacerbates Colitis‐Associated Cancer via IL‐6‐Regulated Macrophage Polarisation and CCL‐20/CCR‐6‐Mediated Lymphocyte Recruitment,” Nature Communications 9, no. 1 (2018): 1646, 10.1038/s41467-018-03773-0. PMC5916940 29695802

[63] X. Fu , C. Yang , and B. Yu , “Targeting KDM5 Demethylases: Inhibition and Degradation,” Current Topics in Medicinal Chemistry 20, no. 4 (2020): 261–263, 10.2174/156802662004200304124340. 32238126

[64] J. Plch , J. Hrabeta , and T. Eckschlager , “KDM5 Demethylases and their Role in Cancer Cell Chemoresistance,” International Journal of Cancer 144, no. 2 (2019): 221–231, 10.1002/ijc.31881. 30246379

[65] B. E. Collins , C. B. Greer , B. C. Coleman , and J. D. Sweatt , “Histone H3 Lysine K4 Methylation and its Role in Learning and Memory,” Epigenetics & Chromatin 12, no. 1 (2019): 7, 10.1186/s13072-018-0251-8. 30616667 PMC6322263

[66] Y. Sato , T. Yoshizato , Y. Shiraishi , et al., “Integrated Molecular Analysis of Clear‐Cell Renal Cell Carcinoma,” Nature Genetics 45, no. 8 (2013): 860–867, 10.1038/ng.2699. 23797736

[67] H. Guak , M. Weiland , A. V. Ark , et al., “Transcriptional Programming Mediated by the Histone Demethylase KDM5C Regulates Dendritic Cell Population Heterogeneity and Function,” Cell Reports 43, no. 8 (2024): 114506, 10.1016/j.celrep.2024.114506. 39052479 PMC11416765

[68] L. Wu , J. Cao , W. L. Cai , et al., “KDM5 Histone Demethylases Repress Immune Response via Suppression of STING,” PLoS Biology 16, no. 8 (2018): 2006134, 10.1371/journal.pbio.2006134. PMC6095604 30080846

[69] G. Galliverti , S. Wullschleger , M. Tichet , et al., “Myeloid Cells Orchestrate Systemic Immunosuppression, Impairing the Efficacy of Immunotherapy Against HPV+ Cancers,” Cancer Immunology Research 8, no. 1 (2020): 131–145, 10.1158/2326-6066.CIR-19-0315. 31771984 PMC7485376

[70] F. A. Wolf , P. Angerer , and F. J. Theis , “SCANPY: Large‐Scale Single‐Cell Gene Expression Data Analysis,” Genome Biology 19, no. 1 (2018): 15, 10.1186/s13059-017-1382-0. 29409532 PMC5802054

[71] S. Jin , C. F. Guerrero‐Juarez , L. Zhang , et al., “Inference and Analysis of Cell‐Cell Communication Using CellChat,” Nature Communications 12, no. 1 (2021): 1088, 10.1038/s41467-021-21246-9. PMC7889871 33597522

[72] M. Efremova , M. Vento‐Tormo , S. A. Teichmann , and R. Vento‐Tormo , “CellPhoneDB: Inferring Cell–Cell Communication From Combined Expression of Multi‐Subunit Ligand–Receptor Complexes,” Nature Protocols 15, no. 4 (2020): 1484–1506, 10.1038/s41596-020-0292-x. 32103204

[73] X. Qiu , A. Hill , J. Packer , D. Lin , Y. A. Ma , and C. Trapnell , “Single‐Cell mRNA Quantification and Differential Analysis with Census,” Nature Methods 14, no. 3 (2017): 309–315, 10.1038/nmeth.4150. 28114287 PMC5330805

[74] B. Van de Sande , C. Flerin , K. Davie , et al., “A Scalable SCENIC Workflow for Single‐Cell Gene Regulatory Network Analysis,” Nature Protocols 15, no. 7 (2020): 2247–2276, 10.1038/s41596-020-0336-2. 32561888

[75] I. Tirosh , A. S. Venteicher , C. Hebert , et al., “Single‐Cell RNA‐Seq Supports a Developmental Hierarchy in Human Oligodendroglioma,” Nature 539, no. 7628 (2016): 309–313, 10.1038/nature20123. 27806376 PMC5465819

[76] A. Dobin , C. A. Davis , F. Schlesinger , et al., “STAR: Ultrafast Universal RNA‐Seq Aligner,” Bioinformatics 29, no. 1 (2013): 15–21, 10.1093/bioinformatics/bts635. 23104886 PMC3530905

[77] G. H. Putri , S. Anders , P. T. Pyl , J. E. Pimanda , and F. Zanini , “Analysing High‐Throughput Sequencing Data in Python with HTSeq 2.0,” Bioinformatics 38, no. 10 (2022): 2943–2945, 10.1093/bioinformatics/btac166. 35561197 PMC9113351

[78] B. Langmead and S. L. Salzberg , “Fast Gapped‐Read Alignment with Bowtie 2,” Nature Methods 9, no. 4 (2012): 357–359, 10.1038/nmeth.1923. 22388286 PMC3322381

[79] P. Danecek , J. K. Bonfield , J. Liddle , et al., “Twelve Years of SAMtools and BCFtools,” Gigascience 10, no. 2 (2021): giab008, 10.1093/gigascience/giab008. 33590861 PMC7931819

[80] Y. Zhang , T. Liu , C. A. Meyer , et al., “Model‐Based Analysis of ChIP‐Seq (MACS),” Genome Biology 9, no. 9 (2008): R137, 10.1186/gb-2008-9-9-r137. 18798982 PMC2592715

[81] F. Ramirez , D. P. Ryan , B. Gruning , et al., “deepTools2: A Next Generation Web Server for Deep‐Sequencing Data Analysis,” Nucleic Acids Research 44, no. W1 (2016): W160–W165, 10.1093/nar/gkw257. 27079975 PMC4987876

[82] A. R. Quinlan and I. M. Hall , “BEDTools: A Flexible Suite of Utilities for Comparing Genomic Features,” Bioinformatics 26, no. 6 (2010): 841–842, 10.1093/bioinformatics/btq033. 20110278 PMC2832824

[83] J. T. Robinson , H. Thorvaldsdottir , W. Winckler , et al., “Integrative Genomics Viewer,” Nature Biotechnology 29, no. 1 (2011): 24–26, 10.1038/nbt.1754. PMC3346182 21221095

[84] S. Zhang , X. Chen , E. Jin , et al., “The GSA Family in 2025: A Broadened Sharing Platform for Multi‐Omics and Multimodal Data,” Genomics, Proteomics & Bioinformatics 23, no. 4 (2025): qzaf072, 10.1093/gpbjnl/qzaf072. PMC12451262 40857552

[85] C.‐N. Members , “Partners,” Nucleic Acids Research 53, no. D1 (2025): D30–D44, 10.1093/nar/gkae978. 39530327 PMC11701749

