# Supplementary material for: PBRM1 Deficiency Reshapes an Immune Suppressive Microenvironment Through Epigenetic Tuning of PBRM1‐KDM5C‐IL6 Axis in ccRCC
Source: Adv Sci (Weinh). 2026 Jan 9;13(16):e12627. doi: 10.1002/advs.202512627 (PMC13042695; doi:10.1002/advs.202512627)
Supplement: Supplementary file 1 — Supporting File 1: advs73755‐sup‐0001‐SuppMat.docx. [file ADVS-13-e12627-s003.docx]

**Supplementary Material**

**Supplementary Figures:**


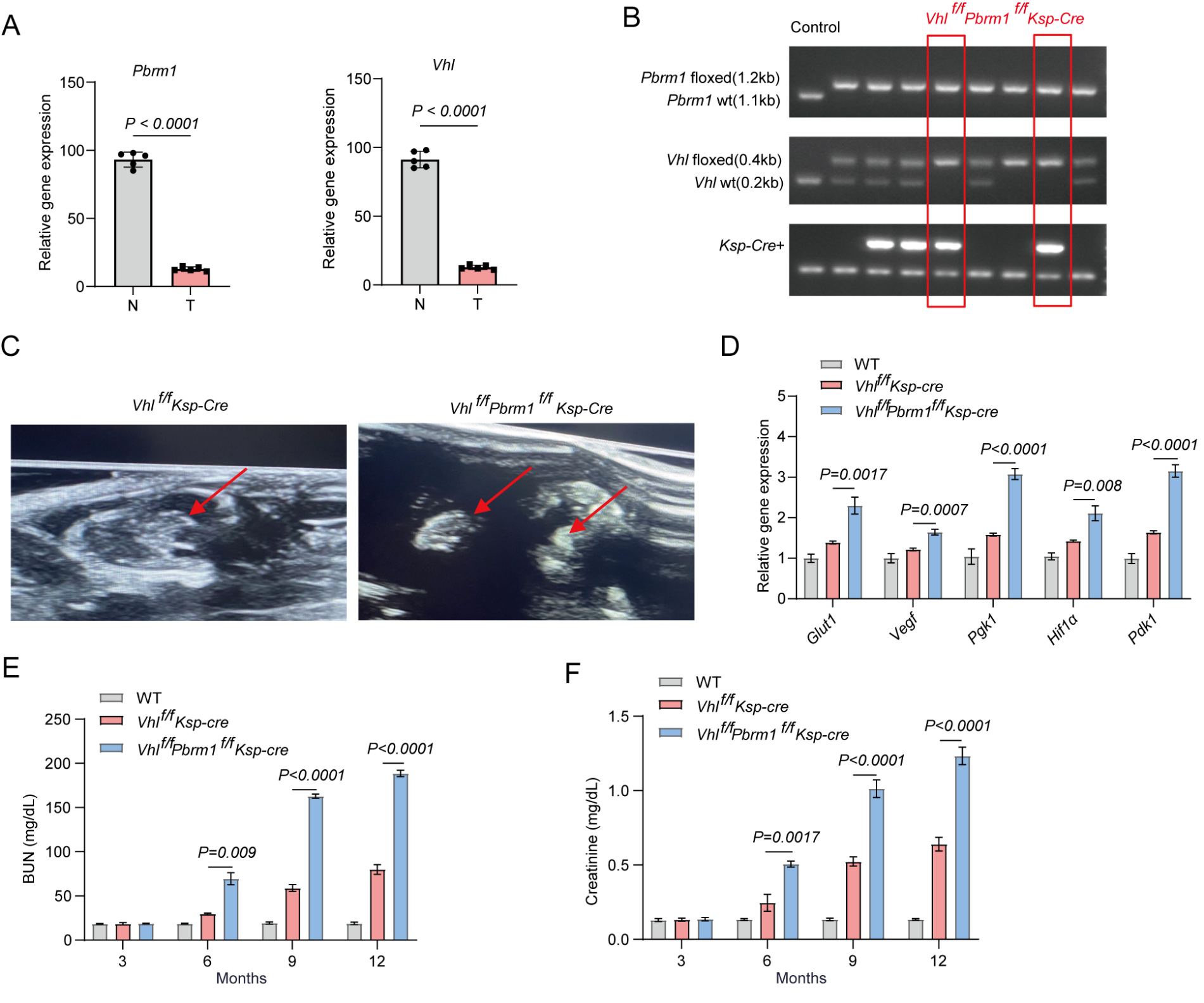


**Supplementary Figure S1.** ***Vhl*^f/f^*Pbrm1*^f/f^*Ksp*-*Cre* Mice Develop Multifocal ccRCC**.

(A) qRT-PCR analysis of *Vhl* and *Pbrm1* mRNA levels in the kidneys of control and *Vhl^f/f^Pbrm1^f/f^Ksp-Cre* mice. (B) qRT-PCR genotyping of DNA obtained from kidney of control and *Vhl*^f/f^*Pbrm1*^f/f^*Ksp*-*Cre* mice. (C) Comparison of sizes between *Vhl^f/f^Ksp-Cre* and *Vhl^f/f^Pbrm1^f/f^Ksp-Cre* mice. (D) qRT-PCR for the indicated HIF-1α target genes in kidneys of WT, *Vhl^f/f^Ksp-Cre* mice and *Vhl^f/f^Pbrm1^f/f^Ksp-Cre* mice. (E) Plasma BUN measurements in WT, *Vhl^f/f^Ksp-Cre* mice and *Vhl^f/f^Pbrm1^f/f^Ksp-Cre* mice. (F) Plasma creatinine measurements in WT, *Vhl^f/f^Ksp-Cre* mice and *Vhl^f/f^Pbrm1^f/f^Ksp-Cre* mice. N=5, Data presented as Mean ± SEM, unpaired two-tailed Student’s t test (A), one-way ANOVA (D, E and F). Data are representative of three independent experiments with similar results.


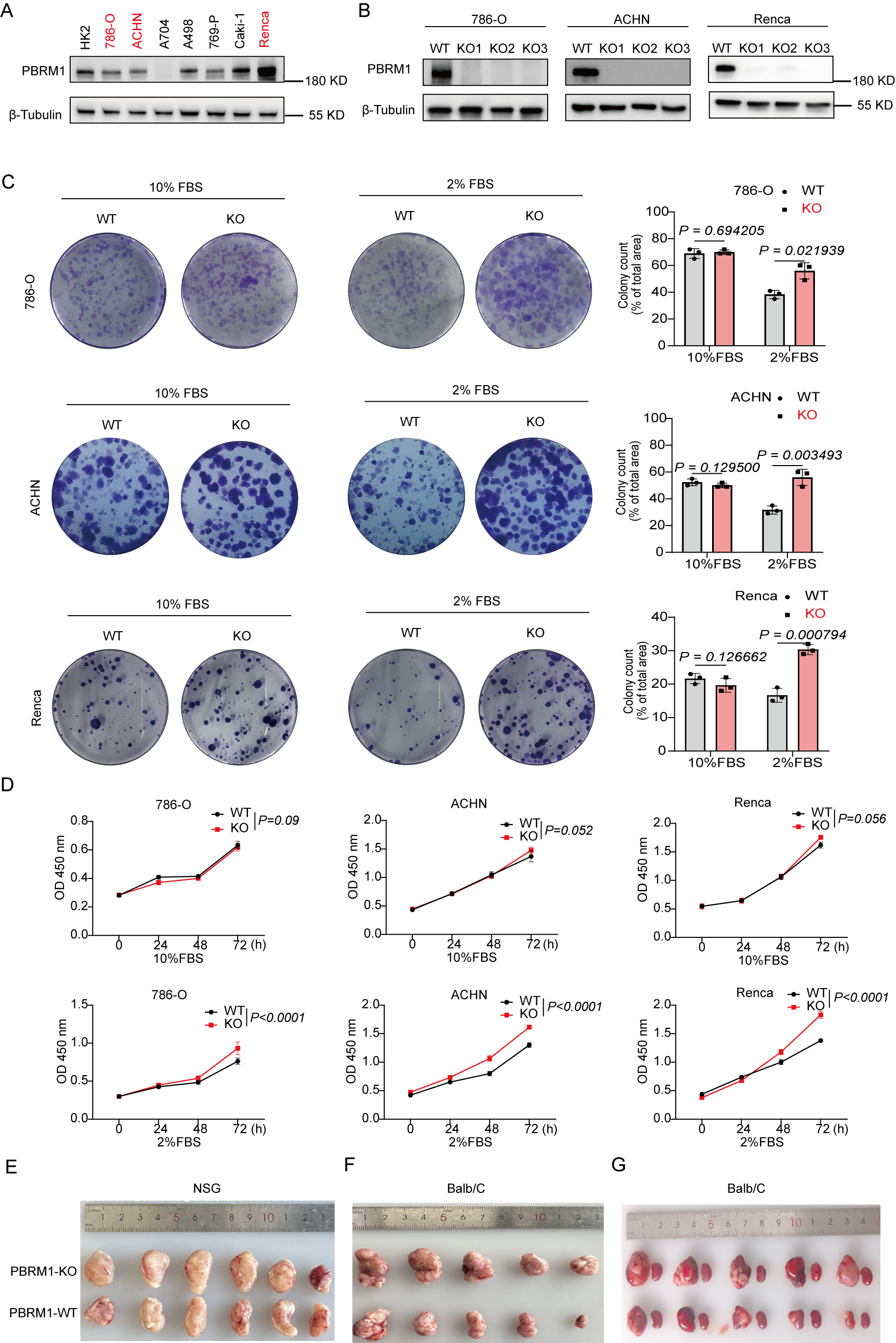


**Supplementary Figure S2.** **PBRM1 loss is pro-tumorigenic**.

(A) Western blot analysis of the PBRM1 proteins in normal kidney epithelial cell lines and ccRCC cell lines. (B) Protein levels of PBRM1 in three sets of isogenic cell lines, in which we knocked out PBRM1. Relative quantified PBRM1 protein levels normalized to β-Tubulin. (C) In vitro colony formation (measured as the percentage of total plate area) of three isogenic cell lines under 10% or low serum (2%) (n = 3). (D) In vitro cell proliferation of three isogenic cell lines under 10% or low serum (2%) (n = 6). (E) In vivo tumor formation of 786-O PBRM1 WT and 786-O PBRM1 KO cells in NSG mice (n = 6). (F) In vivo tumor formation of Renca *Pbrm1* WT and Renca *Pbrm1* KO cells in Balb/C mice (n = 5). (G) In vivo of the orthotopic ccRCC tumors formation of Renca *Pbrm1* WT and Renca *Pbrm1* KO cells in Balb/C mice (n = 5). Data presented as Mean ± SEM, one-way ANOVA (C), and unpaired two-tailed Student’s t test (D). Data are representative of three independent experiments with similar results.


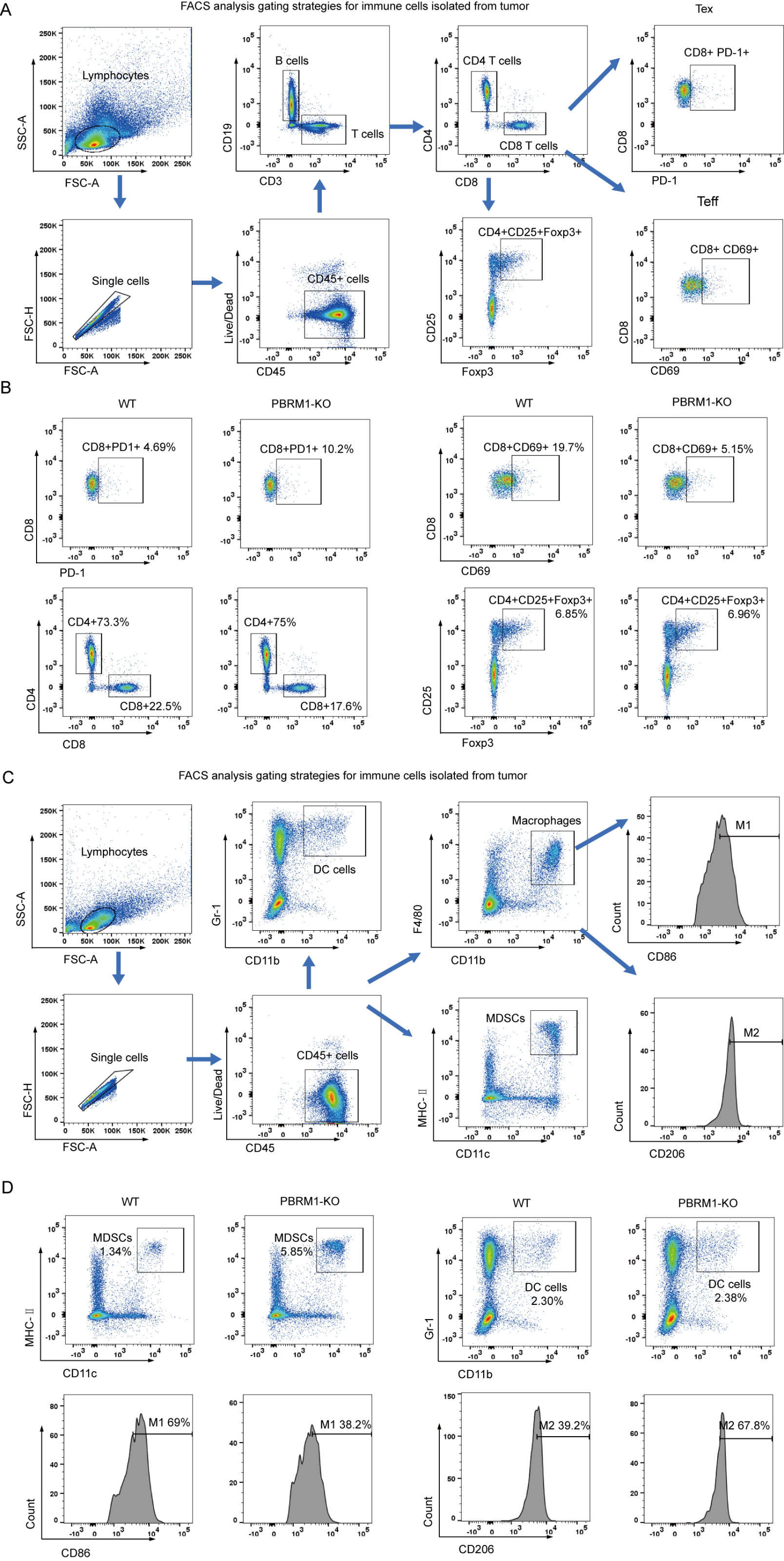


**Supplementary Figure S3.** **Characteristics of immune cells in ccRCC**.

(A) Gating strategies for flow cytometry analysis of tumors used in this study. (B) Representative flow cytometry plots for T cells and B cells in WT and Pbrm1 KO ccRCC tissues. (C) FACS analysis gating strategies for immune cells isolated from ccRCC tissue. (D) Representative flow cytometry plots for DCs, MDSCs and macrophages in WT and Pbrm1 KO ccRCC tissues.


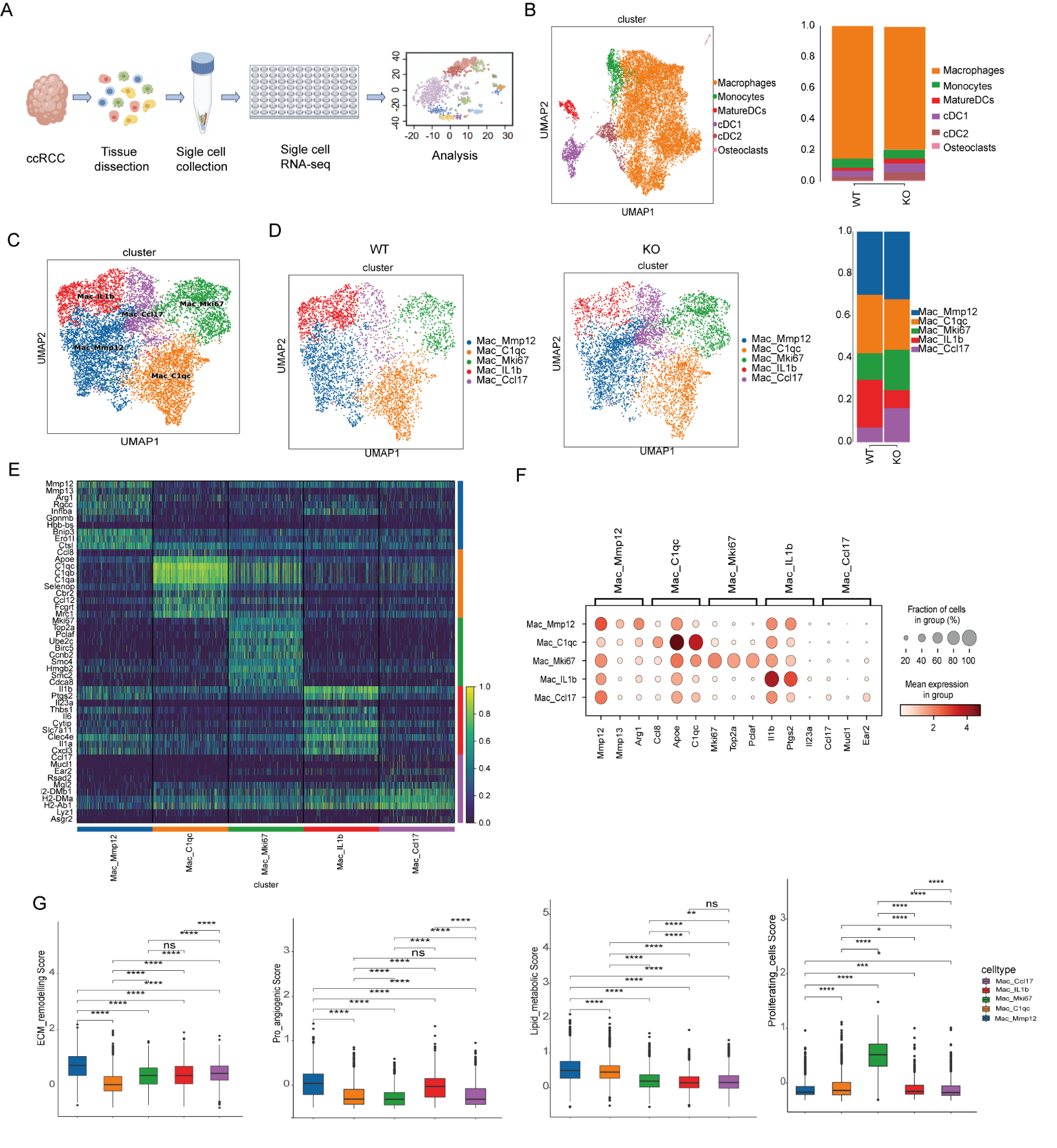


**Supplementary Figure S4.** **scRNA-seq analysis identifies a distinct macrophage subset in Pbrm1-deficient ccRCC tumors**.

(A) Graphic overview of this study design. WT and Pbrm1 KO tumors were processed into single-cell suspension and unsorted cells were used for scRNA-seq. (B) UMAP representation and graph-based clustering of merged scRNA-seq data from all groups. (C) Reclustering of macrophage displayed in a UMAP plot, with circled five Mac subclusters. (D) Relative cell proportion of macrophage subclusters in WT and Pbrm1 KO tumors. (E) Heatmap showing the expression of marker genes in the indicated cell types. (F) Dot plots showing average expression of known markers in indicated cell clusters. The dot size represents percent of cells expressing the genes in each cluster. The expression intensity of markers is shown. (G) Similarity of de novo ssGSEA annotated programs compared to previously reported macrophage signatures. Data presented as Mean ± SEM, one-way ANOVA (G).


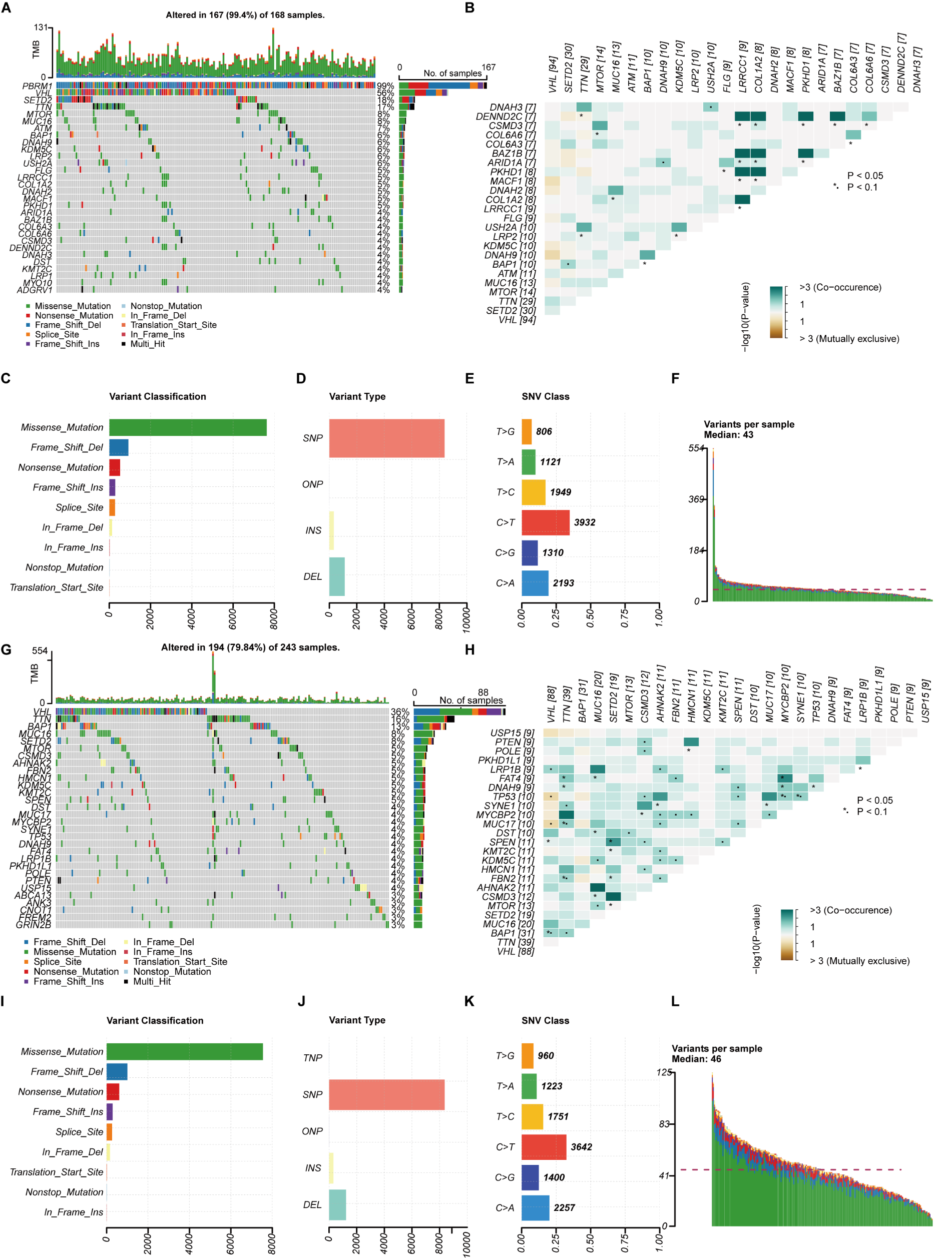


**Supplementary Figure S5.** **Mutational summary of PBRM1-mutated and PBRM1-wildtype ccRCC cohorts from the TCGA database.** (A, G) Oncoplots delineate mutation frequencies in both PBRM1-mutated (A) and PBRM1-WT (G) ccRCC cohorts. (B, H) Triangular heatmaps visualize the co-occurrence and mutual exclusivity patterns of gene mutations across PBRM1-mutated (B) and PBRM1-WT (H) ccRCC cohorts. (C, I) Bar charts summarize gene variant classifications for all patients within the PBRM1-mutated (C) and PBRM1-WT (I) ccRCC cohorts. (D, J) Bar charts specify the distribution of gene variant types within the PBRM1-mutated (D) and PBRM1-WT (J) ccRCC cohorts. (E, K) Bar charts focus on the classification of single nucleotide variants (SNVs) within the PBRM1-mutated (E) and PBRM1-WT (K) ccRCC cohorts.

(F, L) Bar charts quantify the number of mutations per patient in the PBRM1-mutated (F) and PBRM1-WT (L) ccRCC cohorts.


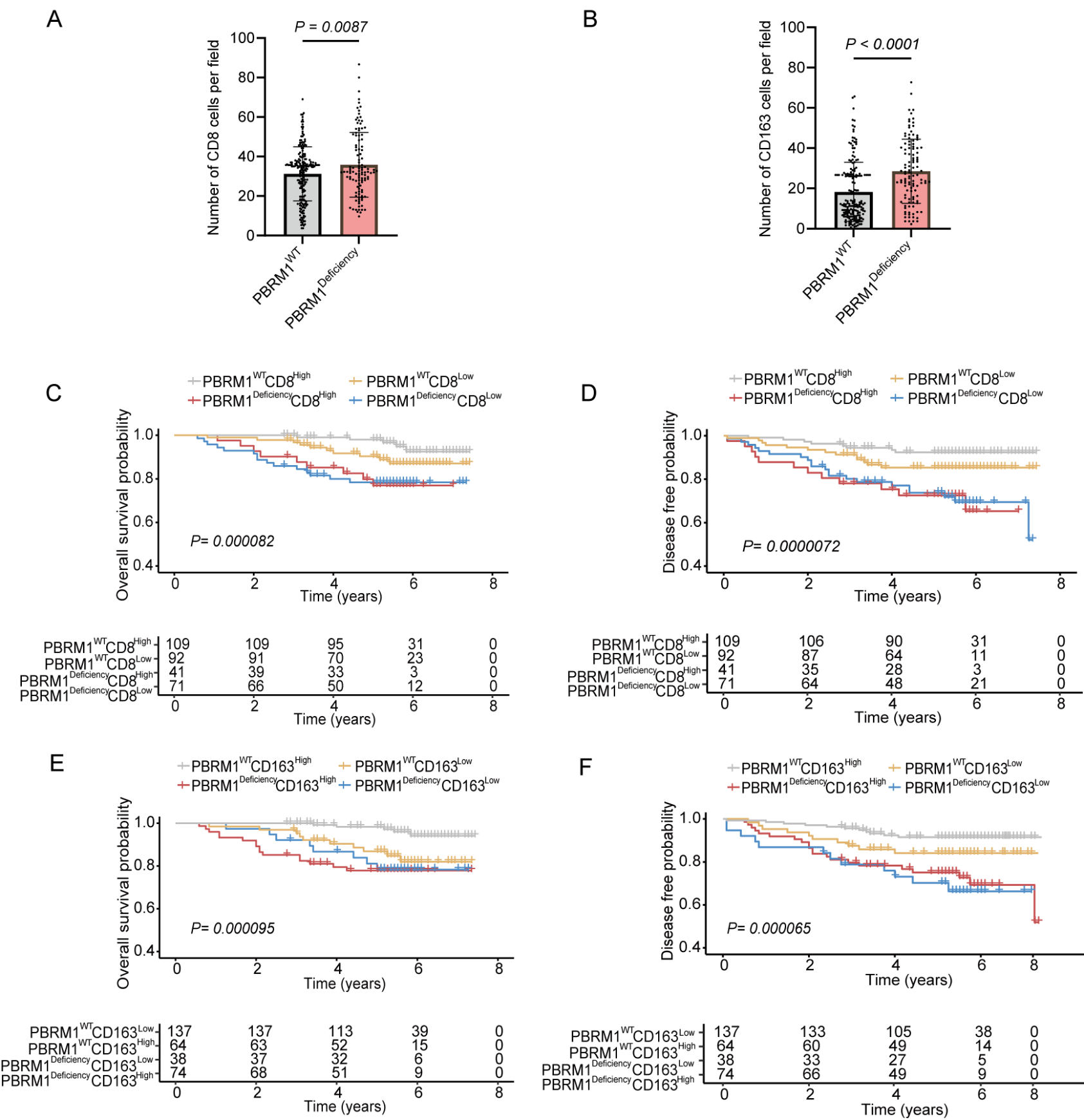


**Supplementary Figure S6.** **Expression of PBRM1, CD8 and CD163 in ccRCC tissues correlates with each other and with the prognosis of ccRCC patients.** (A) Quantification of CD8 expression in PBRM1-WT or PBRM1-deficient ccRCC patient cohort. (B) Quantification of CD163 expression in PBRM1-WT or PBRM1-deficient ccRCC patient cohort. (C) OS curves comparison between the indicated ccRCC patient cohorts with PBRM1/CD8 co-expression using the Mantel-Cox test. (D) DFS curves comparison between the indicated ccRCC patient cohorts with PBRM1/CD8 co-expression using the Mantel-Cox test. (E) OS curves comparison between the indicated ccRCC patient cohorts with PBRM1/CD163 co-expression using the Mantel-Cox test. (F) DFS curves comparison between the indicated ccRCC patient cohorts with PBRM1/CD163 co-expression using the Mantel-Cox test. Data presented as Mean ± SEM, unpaired two-tailed Student’s t test (A) (B), Mantel-Cox test in (C-K). Data are representative of three independent experiments with similar results.


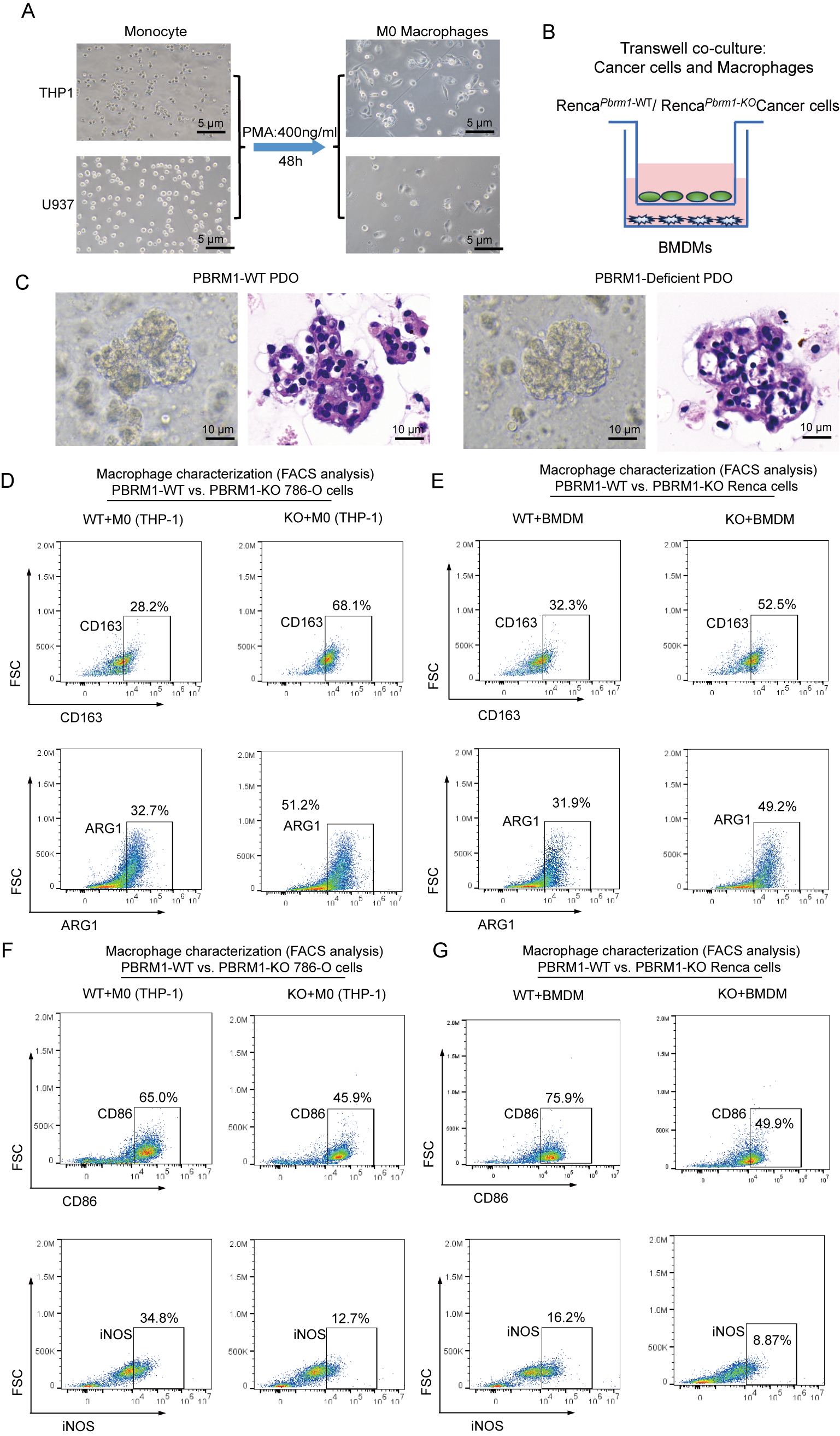


**Supplementary Figure S7.** **IL-6 facilitates chemotactic migration, M2-like polarization of macrophages and upregulation of PD-L1**. (A) Morphological changes (from suspended cells to antennal, spindle-shaped adherent cells) on the induction of monocyte cells to M0 macrophages. scale bar, 50 μm (n=6). (B) Schematic representation of a trans-well coculture assay involving ex vivo programmed M0s combined with WT and PBRM1 KO cancer cells. scale bar, 50 μm (n=6). (C) Representative pictures of clear cell ccRCC PDO cells and HE pictures of ccRCC PDO cells. (D) FACS analysis for ARG1, CD163 (M2 TAM-like marker) protein expression for M0 (THP-1) cocultured with WT and PBRM1 KO cancer cells (786-O). (E) FACS analysis for ARG1, CD163 (M2 TAM-like marker) protein expression for M0 (BMDM) cocultured with WT and Pbrm1 KO cancer cells (Renca). (F) FACS analysis for CD86 and iNOS (M1 TAM-like marker) protein expression for M0 (THP-1) cocultured with WT and PBRM1 KO cancer cells (786-O). (G) FACS analysis for CD86 and iNOS (M1 TAM-like marker) protein expression for M0 (BMDM) cocultured with WT and Pbrm1 KO cancer cells (Renca).


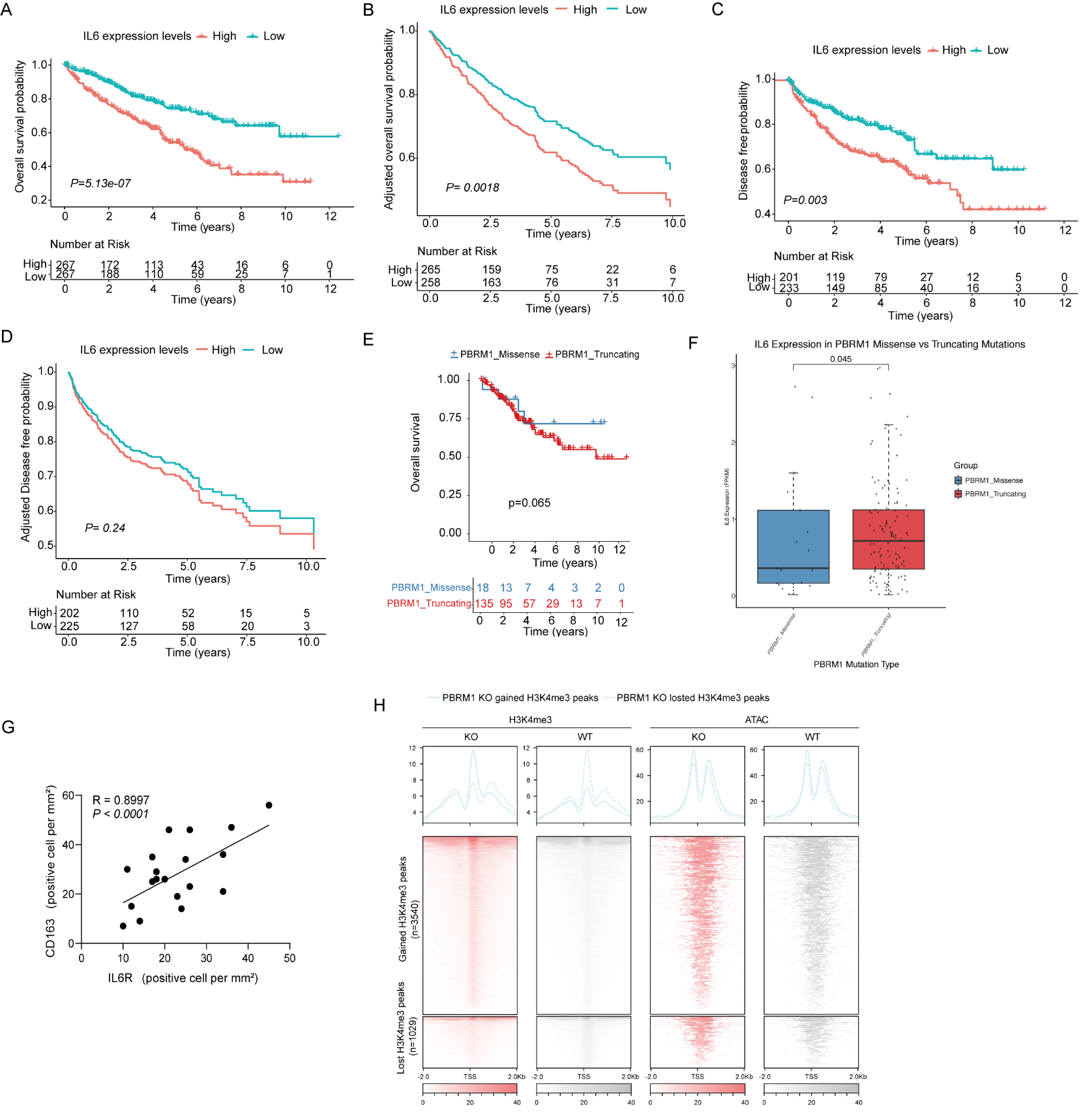


**Supplementary Figure S8.** **IL-6 was positively correlated with CD163^+^ M2-like macrophages in ccRCC**. (A) OS curves comparison between the ccRCC patients with high or low expression of IL-6 using the Mantel-Cox test. (B) OS curves comparison between the ccRCC patients with high or low expression of IL-6 using the Mantel-Cox test with the adjusted Curves R package. (C) DFS curves comparison between the ccRCC patients with high or low expression of IL-6 using the Mantel-Cox test. (D) DFS curves comparison between the ccRCC patients with high or low expression of IL-6 using the Mantel-Cox test with the adjusted Curves R package. (E) OS curves comparison between the ccRCC patients with PBRM1 missense and truncating. (F) The expression of IL-6 in ccRCC patients with PBRM1 missense and truncating. (G) The expression of IL6R was positively correlated with CD163^+^ M2 macrophages in ccRCC tissues. R, Pearson correlation coefficient. (H) H3K4me3 ChIP-seq track showing an example of a gained H3K4me3 peak in both PBRM1 KO lines (red) compared to the control line (gray).


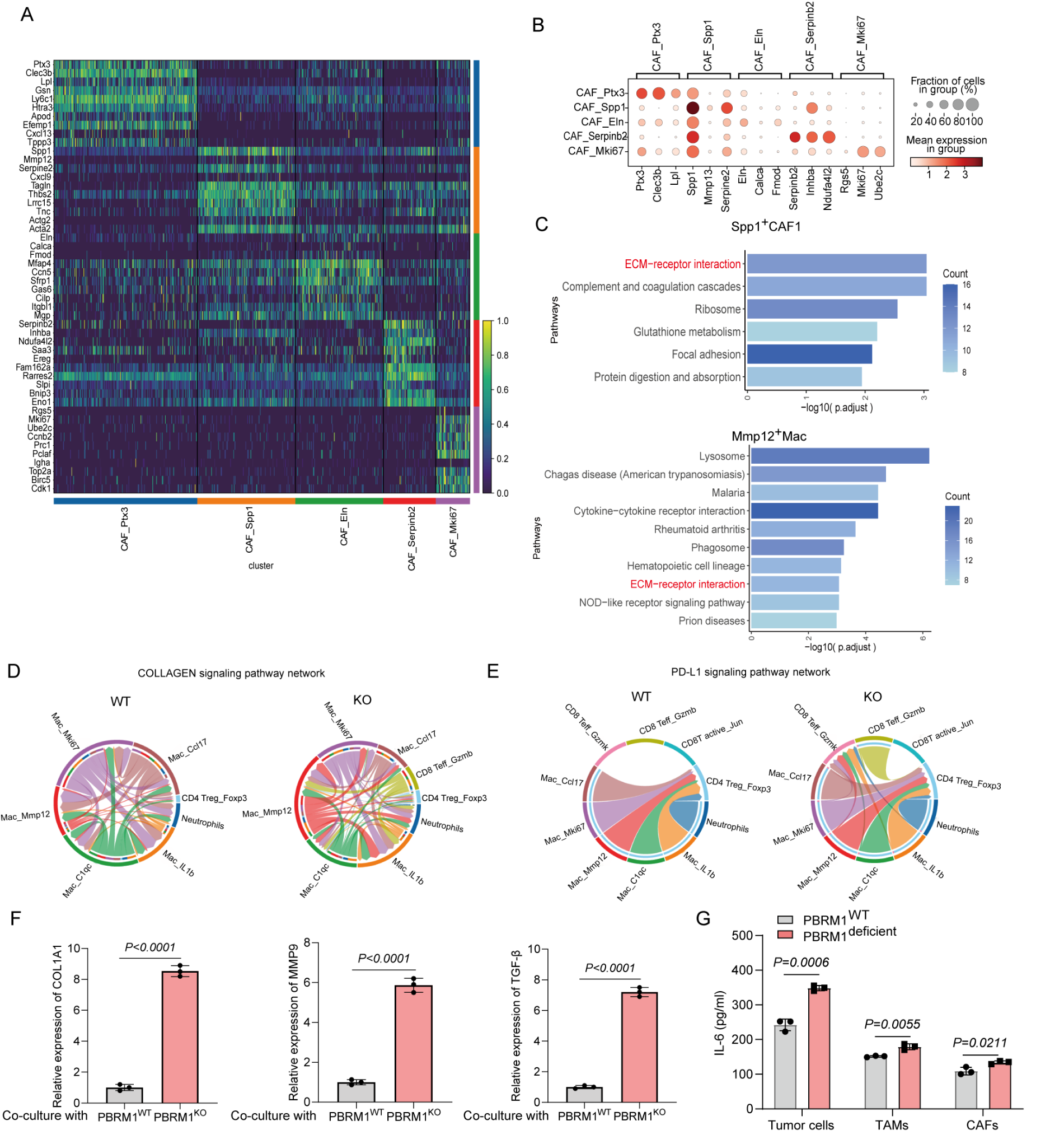


**Supplementary Figure S9.** **scRNA-seq analysis of CAFs subsets in *Pbrm1*-deficient ccRCC**. (A) Heatmap showing the expression of marker genes in the indicated cell types. (B) Re-clustering of CAFs displayed in a dot plot. (C) Pathway enrichment analysis of the upregulated genes in Mmp12^+^ macrophages and Spp1^+^ CAFs. (D) Inferred interactions of COLLAGEN signaling network between macrophages and T cells quantified by Cell Chart. (E) Inferred interactions of PD-L1 signaling network between macrophages and T cells quantified by Cell Chart. (F) qRT-PCR of mRNA expression COL1A1, MMP9, TGF-β in CAFs cocultured with WT and PBRM1 KO cancer cells. (G) ELISA analysis of IL-6 expression in tumor cells, TAMs and CAFs.
