# Supplementary material for: PBRM1 Deficiency Reshapes an Immune Suppressive Microenvironment Through Epigenetic Tuning of PBRM1‐KDM5C‐IL6 Axis in ccRCC
Source: Adv Sci (Weinh). 2026 Jan 9;13(16):e12627. doi: 10.1002/advs.202512627 (PMC13042695; doi:10.1002/advs.202512627)
Supplement: Supplementary file 11 — Supporting File 11: advs73755‐sup‐0011‐TableS10.docx. [file ADVS-13-e12627-s011.docx]

**Antibodies and Reagents**

| REAGENT or RESOURCE | SOURCE | IDENTIFIER |
| --- | --- | --- |
| Antibodies |  |  |
| Rabbit anti-PBRM1 antibody | CST | Cat#89123S |
| Rabbit anti-IL-6 antibody | CST | Cat#12912S |
| Rabbit anti-β-Tubulin antibody | CST | Cat#2128S |
| Rabbit anti-p-JAK1 antibody | CST | Cat#3331S |
| Rabbit anti-JAK1 antibody | CST | Cat#3344S |
| Rabbit anti-p-STAT3 antibody | CST | Cat#9145S |
| Stat3 (D3Z2G) Rabbit mAb | CST | Cat#12640S |
| Rabbit anti-KDM5C antibody | Bethyl | Cat#A301-034A |
| Rabbit anti-GAPDH antibody | CST | Cat#5174S |
| MMP-9 Antibody | CST | Cat#3852 |
| COL1A1 (E8I9Z) Rabbit Monoclonal Antibody | CST | Cat#91144 |
| FAP (F1A4G) Rabbit Monoclonal Antibody | CST | Cat#52818 |
| Fixable Viability Stain 510 | BD | Cat#564406 |
| APC-Cy7 Rat Anti-Mouse CD45(30-F11) | BD | Cat#557659 |
| BV650 Rat Anti-Mouse CD45R/B220(RA3-6B2) | BD | Cat#563893 |
| BV650 Rat Anti-Mouse CD19(1D3) | BD | Cat#563235 |
| FITC Rat Anti-Mouse CD3 Molecular Complex(17A2) | BD | Cat#561798 |
| Alexa Fluor 700 Rat Anti-Mouse CD4(RM4-5) | BD | Cat#557956 |
| BV605 Rat Anti-Mouse CD8a(53-6.7) | BD | Cat#563152 |
| BV421 Rat Anti-Mouse CD25(3C7) | BD | Cat#564370 |
| ANTI-M/R FOXP3 FJK-16S PE | Thermo | Cat#12-5773-82 |
| APC Hamster Anti-Mouse CD69(H1.2F3) | BD | Cat#560689 |
| BB700 Hamster Anti-Mouse CD279 (PD-1)(J43) | BD | Cat#566514 |
| BV421 Rat Anti-Mouse CD107a(1D4B) | BD | Cat#564347 |
| ANTI-M GRANZYME B NGZB PE-CYN7 | Thermo | Cat#25-8898-82 |
| PerCP-Cy5.5 Rat Anti-Mouse IFN-γ(XMG1.2) | BD | Cat#560660 |
| BV786 Mouse Anti-Mouse TIGIT(1G9) | BD | Cat#744215 |
| PE-Cy7 Rat Anti-CD11b(M1/70) | BD | Cat#552850 |
| BV650 Rat Anti-Mouse Ly-6G and Ly-6C(RB6-8C5) | BD | Cat#740454 |
| FITC Rat Anti-Mouse I-A/I-E(2G9) | BD | Cat#562009 |
| BB700 Hamster Anti-Mouse CD11C(HL3) | BD | Cat#566504 |
| APC Hamster anti-Mouse CD80(16-10A1) | BD | Cat#560016 |
| PE Rat Anti-Mouse CD86(GL1) | BD | Cat#561963 |
| BV421 Rat Anti-Mouse F4/80(T45-2342) | BD | Cat#565411 |
| Rat Anti-Mouse CD16/CD32 (2.4G2) | BD | Cat#553141 |
| ANTI-Mouse CD163 TNKUPJ FITC | Thermo | Cat#11-1631-82 |
| ANTI-HHUMAN ARG1 AA1EXF5 PE-CYN7 | Thermo | Cat#25-3697-80 |
| APC Mouse Anti-Human CD206 (19.2) | BD | Cat#561763 |
| Hu CD86 BV421 (FUV-1) | BD | Cat#562433 |
| Rabbit Anti-CD163 | abcam | Cat#ab182422 |
| Rabbit Anti-CD8 | abcam | Cat#ab237709 |
| Rabbit Anti-PD1 | abcam | Cat#ab237728 |
| Rabbit Anti-PD-L1 | abcam | Cat#ab205921 |
| Rabbit Anti-FOXP3 | abcam | Cat#ab20034 |
| Rabbit Anti-HIF-1α | abcam | Cat#ab114977 |
| Rabbit Anti-Ki67 | abcam | Cat#ab15580 |
| Rabbit Anti-CD68 | abcam | Cat#ab955 |
| Rabbit Anti-alpha smooth muscle Actin | abcam | Cat#ab150301 |
| Rabbit Anti-Carbonic Anhydrase 9/CA9 | abcam | Cat#ab243660 |
| Rabbit mAb IgG Isotype Control | Ptm-biolab | Cat#PTM-5073 |
| Anti-Histone H3 Rabbit mAb | Ptm-biolab | Cat#PTM-6600 |
| Anti-Tri-Methyl-Histone H3 (Lys4) Mouse mAb | Ptm-biolab | Cat#PTM-5019 |
| Anti-Mono-Methyl-Histone H3 (Lys4) Mouse mAb | Ptm-biolab | Cat#PTM-5158 |
| Anti-Acetyl-Histone H3 (Lys27) Rabbit mAb | Ptm-biolab | Cat#PTM-116RM |
| Critical commercial assays |  |  |
| IP/CoIP Kit | Absin | Cat#abs955-50 |
| ChIP Kit | Absin | Cat#abs50034-50 |
| Mouse tumor dissociation kit | Miltenyi | Cat#130-096-730 |
| Gentle MACS Dissociator | Miltenyi | Cat# 130-093-235 |
| Tumor Cell Isolation Kit, human | Miltenyi | Cat#130-108-339 |
| Anti-Fibroblast MicroBeads, human | Miltenyi | Cat#130-050-601 |
| CD163 MicroBead Kit, human | Miltenyi | Cat#130-124-420 |
| Mycoplasma Detection Kit | TransGen | Cat# FM311-01 |
| Seven-color multiplex fluorescent  immunohistochemical staining kit | Absin | Cat# abs50015 |
| IL-6 ELISA kit | Dakewe | Cat#1110602 |
| GranzymB ELISA kit | Dakewe | Cat#1118502 |
| TNF-α ELISA kit | Dakewe | Cat#1117202 |
| TNF-γ ELISA kit | Dakewe | Cat#1110002 |
| Chemicals, peptides, and recombinants proteins |  |  |
| RNAiso Plus | Takara | Cat# 9108 |
| Penicillin-Streptomycin 100X solution | HyClone | Cat# SV30010 |
| DMEM-high glucose | Cytiva | Cat#SH30003.01 |
| RPMI1640 | KeyGen | Cat#KGL1503 |
| MEM(NEAA) | Procell | Cat#PM150410 |
| PBS | KeyGen | Cat#KGL2206 |
| Fetal Bovine Serum | Gibco | Cat#A5669701 |
| RIPA buffer | Thermo | Cat#89901 |
| PMSF | Beyotime | Cat#ST506 |
| SYBR qPCR Master Mix | Vazyme | Cat#Q321 |
| Leukocyte Activation Cocktail, with BD GolgiPlug | BD | Cat#550583 |
| Fixation/Permeablization Kit | BD | Cat#554714 |
| Recombinant DNA |  |  |
| LentiCRISPRv2 vector | Addgene | Cat#82416 |
| ps.pAX2 vector | Addgene | Cat#12260 |
| pMD.2G vector | Addgene | Cat#12259 |
| pEGFP-1 vector | YouBio | Cat#VT1118 |
| Software and algorithms |  |  |
| FlowJo v10.1 | Tree Star | https://www.flowjo.com/ |
| GraphPad Prism v9.0 | GraphPad Software | https://www.graphpad.com |
| CaseViewer | 3DHISTECH | https://www.3dhistech.com/  solutions/caseviewer/ |
| LightCycler 96 SW 1.1 | Roche | https://lifescience.roche.com/  global/en/products/productcategory/lightcycler.html#4 |
|  |  |  |
